# Geospatial modeling of pre-intervention nodule prevalence of *Onchocerca volvulus* in Ethiopia as an aid to onchocerciasis elimination

**DOI:** 10.1371/journal.pntd.0010620

**Published:** 2022-07-18

**Authors:** Himal Shrestha, Karen McCulloch, Shannon M. Hedtke, Warwick N. Grant

**Affiliations:** 1 Department of Environment and Genetics, School of Agriculture, Biomedicine and Environment, La Trobe University, Bundoora, Australia; 2 WHO Collaborating Centre for Viral Hepatitis, Victorian Infectious Diseases Reference Laboratory, Royal Melbourne Hospital, and Department of Infectious Diseases, University of Melbourne, at the Peter Doherty Institute for Infection and Immunity, Melbourne, Australia; University of Glasgow, UNITED KINGDOM

## Abstract

**Background:**

Onchocerciasis is a neglected tropical filarial disease transmitted by the bites of blackflies, causing blindness and severe skin lesions. The change in focus for onchocerciasis management from control to elimination requires thorough mapping of pre-control endemicity to identify areas requiring interventions and to monitor progress. *Onchocerca volvulus* nodule prevalence in sub-Saharan Africa is spatially continuous and heterogeneous, and highly endemic areas may contribute to transmission in areas of low endemicity or vice-versa. Ethiopia is one such onchocerciasis-endemic country with heterogeneous *O*. *volvulus* nodule prevalence, and many districts are still unmapped despite their potential for onchocerciasis transmission.

**Methodology/Principle findings:**

A Bayesian geostatistical model was fitted for retrospective pre-intervention nodule prevalence data collected from 916 unique sites and 35,077 people across Ethiopia. We used multiple environmental, socio-demographic, and climate variables to estimate the pre-intervention prevalence of *O*. *volvulus* nodules across Ethiopia and to explore their relationship with prevalence. Prevalence was high in southern and northwestern Ethiopia and low in Ethiopia’s central and eastern parts. Distance to the nearest river (RR: 0.9850, 95% BCI: 0.9751–0.995), precipitation seasonality (RR: 0.9837, 95% BCI: 0.9681–0.9995), and flow accumulation (RR: 0.9586, 95% BCI: 0.9321–0.9816) were negatively associated with *O*. *volvulus* nodule prevalence, while soil moisture (RR: 1.0218, 95% BCI: 1.0135–1.0302) was positively associated. The model estimated the number of pre-intervention cases of *O*. *volvulus* nodules in Ethiopia to be around 6.48 million (95% BCI: 3.53–13.04 million).

**Conclusions/Significance:**

Nodule prevalence distribution was correlated with habitat suitability for vector breeding and associated biting behavior. The modeled pre-intervention prevalence can be used as a guide for determining priorities for elimination mapping in regions of Ethiopia that are currently unmapped, most of which have comparatively low infection prevalence.

## Author summary

Areas with unknown onchocerciasis endemicity may pose a threat to eliminating transmission because they may re-introduce onchocerciasis to areas where interventions have been successful. Additionally, because vectors (and thus *Onchocerca volvulus* transmission) have specific ecological requirements for growth and development, changes in these ecological factors due to human activities (deforestation, modification of river flows by dam construction, climate change) might change patterns of parasite transmission and endemicity. To estimate the impact of environmental changes, we must first identify ecological factors determining prevalence. We have employed Bayesian geostatistical modeling to create a nationwide *O*. *volvulus* nodule prevalence map for Ethiopia based on pre-intervention nodule prevalence data and have explored the effect of environmental variables on nodule prevalence. We estimated the number of pre-intervention cases of nodules and associated uncertainty in previously unmapped areas of Ethiopia to identify areas that need additional data to increase the prediction accuracy. Hydrological variables such as distance to the nearest river, precipitation seasonality, soil moisture, and flow accumulation are associated significantly with *O*. *volvulus* nodule prevalence. We show that the spatial distribution of nodule prevalence can be estimated based on ecological data and that predicted prevalence can be used as a guide to prioritize pre-intervention mapping.

## Introduction

Mapping infection prevalence is fundamental for control and elimination because it is used to estimate the disease burden and to design and monitor the impacts of interventions. Often the prevalence data are in the form of point data at different locations and time points, and are aggregated at different administrative levels [1]. However, disease risk is a spatially continuous phenomenon that extends across and beyond administrative borders [2]. In addition, mapping strategies change depending on the intended endpoint of the intervention [3]: when elimination of transmission is the goal, the spatial heterogeneity in disease prevalence has to be quantified accurately so that appropriate interventions can be implemented and, where possible, implementation and monitoring can be informed by the spatial distribution of infection rather than simply along local administrative organizational boundaries. When resources or accessibility to an endemic region are limited, as is the case for many neglected tropical diseases, such thorough data collection may not be possible and methods to extrapolate likely prevalence would be useful.

Using geostatistical modeling techniques, point prevalence data can be transformed into a continuous spatial prevalence map of varying endemicity [2,4], rather than reporting binary categorization of areas as endemic or non-endemic [5]. These continuous maps can extrapolate the prevalence measures to previously unmapped regions based on the spatial autocorrelation between the prevalence measures and the influence of known ecological and socio-demographic factors. In addition, geostatistical models provide unbiased quantification of the uncertainty associated with the prevalence estimates.

Onchocerciasis is a neglected tropical disease caused by infection with a filarial nematode, *Onchocerca volvulus*, that is transmitted by the bites of blackflies (*Simulium* spp.). The vectors have a specific ecological niche: they breed around fast-flowing rivers, requiring high aeration and oxygen content for larval development [6]. The flies show diurnal activity and bite humans living in communities near these rivers [7–9]. If the biting blackfly carries the infective stage of the parasite (the 3^rd^ stage larvae, or iL3), the larva leaves the blackfly and enters the human host. Inside the human body, the larva develops into an adult worm and forms a nodule, generally localized subcutaneously. People living with onchocerciasis show a range of chronic clinical manifestations, including onchodermatitis, severe itching, rashes, and visual impairment that may culminate in blindness [10]. More recently, it has also been linked with epilepsy and nodding syndrome in children [11, 12].

Onchocerciasis is currently targeted for elimination via community-directed mass drug administration with ivermectin (MDAi), either annually, semi-annually, or, in some areas, up to four times a year [13]. *Onchocerca volvulus* infection prevalence is measured using counts of microfilariae (mf) in a small skin biopsy (skin snipping), physical examination for the presence of nodules (nodule palpitation), or antibody tests that detect the presence of antibodies against the parasite Ov16 antigen [14]. Rapid Epidemiological Mapping of Onchocerciasis (REMO) uses nodule palpation in combination with geographic information system mapping, and was used by the African Programme for Onchocerciasis Control (APOC) to map prevalence in twenty countries from 1996 to 2012 [15,16]. REMO revealed that the prevalence of *O*. *volvulus* nodules was patchy and heterogenous across Africa [17] and identified areas for ivermectin intervention [3,15] using a threshold for treatment set at a nodule prevalence of 20%. Onchocerciasis-endemic communities were divided into hypoendemic (nodule prevalence: < 20%), meso-endemic (nodule prevalence: 20–45%), and hyperendemic (nodule prevalence: > 45%) [17,18] based on nodule prevalence. However, there are still many areas that are unmapped and in which the nodule prevalence is not known [19].

In onchocerciasis-endemic Ethiopia, mapping of prevalence has been focused on the western districts based on the high incidence of onchocerciasis and because environmental factors favor blackfly breeding in these regions [20]. In contrast, eastern Ethiopia has been assumed to be free of *O*. *volvulus* infection, which has generally proven true [21]. However, a recent continent-level mapping [19] found that most of the implementation units that were predicted to be suitable for onchocerciasis in Ethiopia were not mapped, posing a risk to elimination goals. In addition, there is high spatial variability of onchocerciasis endemicity in Ethiopia, with prevalence ranging from 0% in some areas to as high as 84% in some areas of southwest Ethiopia [21,22].

MDAi started in some Ethiopian hyperendemic foci in 2002 [20] and, to our knowledge, there has not been coordinated vector control in Ethiopia. The shift to onchocerciasis elimination officially began in 2013 with a goal to eliminate transmission by 2020 [20,22]: the program moved from annual to biannual treatment strategy in all the known endemic areas and scaled up treatment to other additional endemic areas which were not treated previously [22]. Cross-border coordination of MDAi between transmission foci in northwestern Ethiopia and bordering Sudan is ongoing [13]. In some cases, transmission decline without intervention has also been reported [23] but onchocerciasis persists in some areas despite MDAi for a variety of reasons, including challenges with treatment compliance [24–26], civil unrest [27, 28], and lately the COVID-19 pandemic [29]. In addition, there has been variation in the history and the frequency of MDAi. Most of the hyper- and mesoendemic districts have been treated over two decades, while in hypoendemic districts, MDAi started around 2014 following the policy shift from control to elimination [22].

There is no national-level baseline endemicity map of *O*. *volvulus* nodule prevalence for Ethiopia, which has created difficulty in quantifying the effect of MDAi on a national scale. Baseline/pre-control endemicity is an important indicator of morbidity and a predictor for the time required for elimination [18,30–32]. In addition, the prevalence measures before intervention provide an unbiased relationship between the infection prevalence and environmental variables. Prevalence and onchocerciasis suitability mapping for Ethiopia in previous studies [17,19] have been done as part of continental-scale research, although Zouré et al. [17] did not consider environmental factors, and Cromwell et al. [19] used presence-absence data which do not capture the magnitude of the prevalence. Although these studies helped to place *O*. *volvulus* infection prevalence or risk in a broader ecological and epidemiological context, we have focused on a spatial scale which offers us greater flexibility to explore ecological patterns unique to Ethiopia by incorporating both the magnitude of prevalence and associated ecological variables [33]. We develop a geostatistical model for the distribution of pre-intervention nodule prevalence of *O*. *volvulus* in Ethiopia using an approach that considers spatial variation in environmental and socio-demographic variables. Furthermore, we identify the most important environmental and socio-demographic variables contributing to *O*. *volvulus* nodule prevalence, and present estimates of uncertainty in the predicted prevalence that can be used to target areas for further mapping efforts.

## Methods

### Prevalence data

*Onchocerca volvulus* infection prevalence data with site-specific coordinates for Ethiopia were obtained from the publicly available Expanded Special Project for Elimination of Neglected Tropical Diseases (ESPEN) database [34]. Nodule prevalence data were collected as part of REMO mapping before the initiation of MDAi, between the years 2001 and 2012, examining the presence of palpable onchocercal nodules in 30 to 50 adults randomly selected from each surveyed village [15,20]. The protocols for the REMO assessment are available in the published guidelines [35]. There were 927 geopositioned coordinates for nodule prevalence in 36,010 people. Any observations from the same geographic coordinates at different times were aggregated by adding the number of cases observed and the number of total tests done before calculating the prevalence.

**Fig 1:**
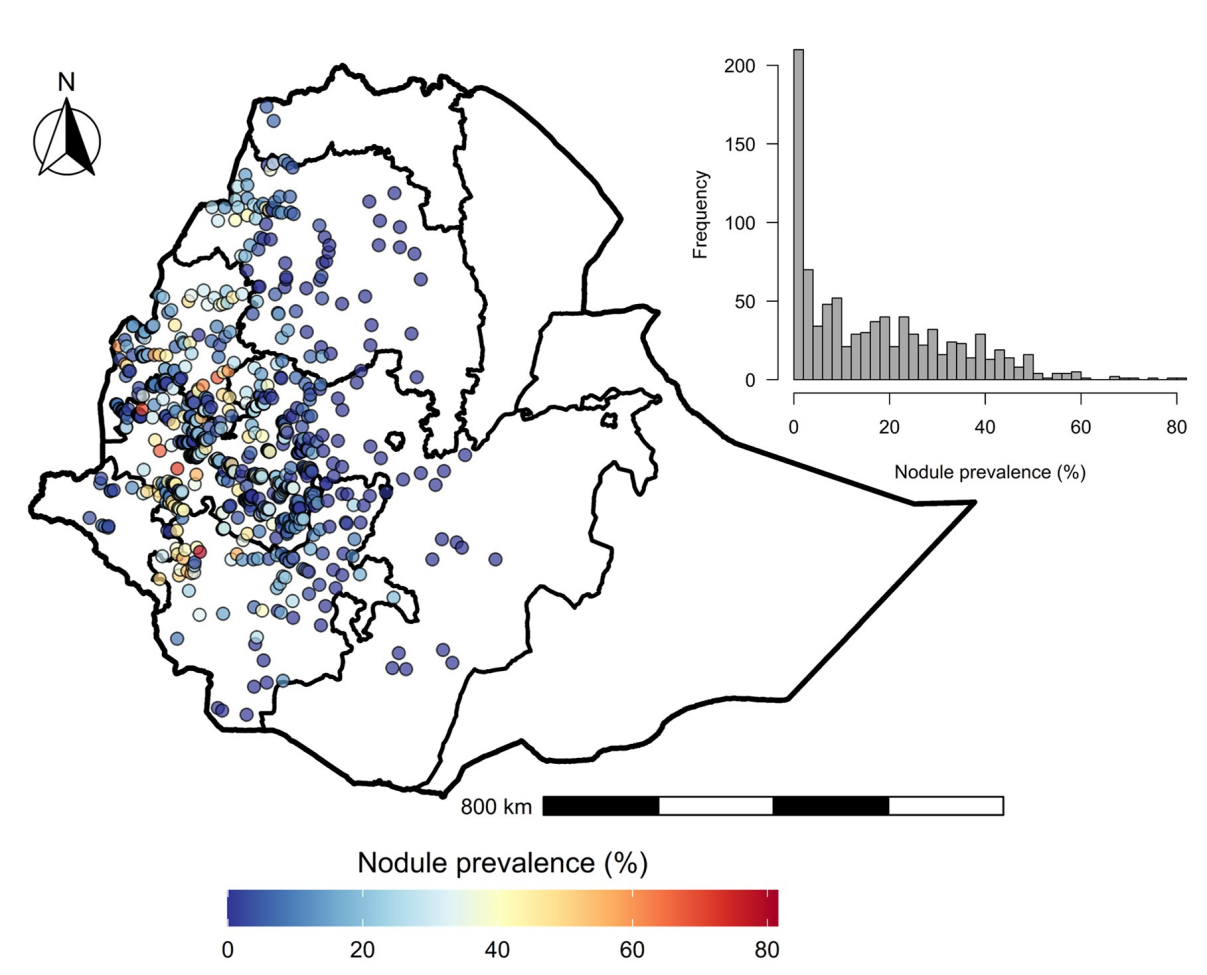
Sites and the nodule prevalence measured during Rapid Epidemiological Mapping of Onchocerciasis (REMO) in Ethiopia. The internal boundaries on the map represents administrative regions. The inset figure shows the histogram of prevalence. The administrative borders are from the Global Administrative Areas (GADM) database (available at: https://gadm.org/maps.html).

Although the database contains both nodule prevalence data and skin mf prevalence data, in this analysis, only nodule prevalence data were considered for the geospatial analysis because of the number of skin mf sampled (n = 126) before MDAi, which limits its utility for identifying associations between prevalence and environmental variables. That said, skin mf data were used to assess the correlation between skin mf as a measure of *O*. *volvulus* infection and the nodule prevalence measure, which revealed two outlier observations with very high nodule prevalence but with low skin mf prevalence (S1 Fig). These were excluded from the dataset because the low skin mf prevalence could not be attributed to a reasonable cause, such as MDAi, as the data were collected before ivermectin distribution. Thus, the final dataset contained nodule prevalence data from 916 unique sites and 35,077 people (Fig 1).

### Environmental, climate, and socio-demographic variables

Variables relevant to *O*. *volvulus* infection prevalence and *Simulium* ecology based on published literature were assembled from different sources and were exported as a raster layer at a resolution of 1 km using Google Earth Engine [6, 36–39] (S1 Table). Raster layers with higher resolution were downsampled using a mean aggregation method, whereas raster layers with lower resolution were resampled to align with 1 km resolution [40] to prepare a raster stack of uniform resolution. Raster data were processed using the *raster* package in R version 4.1.0 [41–43]. Downloaded raster variables were reprojected to a standard projection, World Geodetic System 1984 (WGS84). The raster covariates were cropped to the boundary of Ethiopia (S2 Fig), and a raster stack of covariates was prepared. The measurement of different covariates at each sample site was extracted from the raster stack.

### Variable selection

Thirty-two variables were grouped into six major categories: elevation, temperature, precipitation, socio-demographic, hydrological, and vegetation (S1 Table), and the initial selection of covariates was conducted separately for each category. During the initial rounds of variable selection, multi-collinearity was assessed among the variables by calculating the Pearson correlation coefficient matrix and a variable inflation factor (VIF) for the linear model, including the variables, using the *GGally* and *car* packages in R [44,45]. Next, any variables with an absolute correlation coefficient less than 0.8 with other variables within the group were selected [46]. For the set of covariates with a correlation coefficient greater than 0.8 and a VIF greater than 10, only one of the covariates was selected [33,47]. The VIF measures how easily a given predictor can be predicted from a linear regression based on other predictors. The predictor with the lowest VIF score was selected among the set of correlated covariates. The final covariates yielded a correlation matrix of less than 0.8 (S3 Fig) and a VIF factor of less than 10 (S2 Table). Based on this initial round of analysis, a set of 15 covariates were selected.

Model fit was assessed based on the Deviance Information Criterion (DIC) and Widely Applicable Information Criterion (WAIC) scores [48]. We ran a univariate regression model and calculated the DIC and WAIC scores for the respective univariate models. A covariate yielding the least DIC and WAIC scores from each category was selected. Combinations of other variables were further explored if their inclusion further optimized the model fit scores. Eight covariates from the pool of 15 possible were selected for downstream geostatistical analysis (S3 Table).

### Geo-statistical modelling framework

A Bayesian geostatistical model was implemented using the Integrated Nested Laplace Approximation (INLA) approach, which has been reported to be computationally efficient for posterior distribution calculation and has been employed in recent large-scale geostatistical models [33, 46, 49, 50]. Geostatistical approaches assume a positive spatial correlation between observations; i.e., the observations nearer to each other are more related than the farther ones. Information from neighboring pixels can then be utilized to allow smoothing of extreme values due to small sample sizes and give reliable and robust estimates from sparse data [33, 51]. Further, the hierarchical structure of the model permits the estimation of covariate effects, spatial covariance structure, and the prediction of missing data [33]. These models incorporate both fixed and random effects. The fixed effects determine the influence of covariates on *O*. *volvulus* infection prevalence, while the random effects account for the spatial variation that determines anomalous regions of high and low prevalence [46]. This model can thus identify the relationship between infection prevalence data and several predictors and quantify spatial dependence via the covariance matrix of a Gaussian process facilitated by adding random effects to the observed locations [49].

### Model fitting

Conditional on the true prevalence *P*(*x*_*i*_) at location *x*_*i*_ = 1,2,3….*n*, the number of cases (*Y*_*i*_) observed out of the total number of people tested (*N*_*i*_) were assumed to follow a binomial distribution.


$$\begin{array}{c} Y_{i}|P(x_{i})∼Binomial(N_{i},P(x_{i})) \end{array}$$

The log odds of prevalence is modeled as

$$\begin{array}{c} logit(P(x_{i}))=\beta_{0}+X_{i}^{T}\beta +S(x_{i}). \end{array}$$

where *β*_0_ is the intercept, $X_{i}^{T}$ are the vectors of covariates with their corresponding coefficients *β*. *S*(**x**_*i*_) is a spatial random effect modeled as a zero-mean Gaussian process using the Matérn covariance function which is defined by the equation:

$$\begin{array}{c} \mathrm{Cov}(S(x_{i}),S(x_{j}))=\frac{\sigma^{2}}{2^{\nu -1}\Gamma (\nu )}{\left(\kappa ||x_{i}-x_{j}||\right)}^{\nu }K_{\nu }(\kappa ||x_{i}-x_{j}||). \end{array}$$

Here, *K*_*v*_ is the modified Bessel function of the second kind and order *v*>0, *v* is the smoothness parameter, and *σ*^2^ is the marginal variance [46]. *κ*>0 is the scaling parameter related to the practical range *ρ*, the distance at which the correlation between two points is approximately zero. The empirical definition of range, $=\frac{\sqrt{8\nu }}{\kappa }$, where *ρ* is the distance at which the spatial correlation is close to 0.1, is generally used [2,46,48,52]. Default priors were used for the intercept parameter, effect parameters for the covariates, and the hyperparameters in the model as defined in Moraga, p. 35–37 [2].

### Accounting for excess zero prevalence

The binomial distribution is governed by only a single parameter which does not address overdispersion. To account for the excess zero prevalence in the data (Fig 1), zero-inflated binomial models (ZIB) Type 0 and Type 1 were also considered. There are structural zeros (prevalence reported to be zero based on reality) and sample zeros (prevalence reported to be zero based on chance) in any probability distribution [48,53]. Type 0 model considers only the structural zeros, while Type 1 considers both the structural and sample zeros. With ZIB Type 0 model, the probability density function for the observed cases is

$$\begin{array}{c} p(Y_{i}|P(x_{i}),P_{0})=P_{0}I(Y_{i}=0)+(1-P_{0})I(Y_{i}>0)(\frac{N_{i}}{Y_{i}})P{\left(x_{i}\right)}^{Y_{i}}{\left(1-P(x_{i})\right)}^{N_{i}-Y_{i}} \end{array}$$

Here, *P*_0_ is the proportion of sample zeros, (1−*P*_0_) is the proportion of structural zeros, and *I*(*Y*_*i*_ = 0) is the indicator variable. When both structural zeros and sample zeros are considered, i.e., Type 1, the observations follow the probability density function:

$$\begin{array}{c} p(Y_{i}|P(x_{i}),P_{0})=P_{0}I(Y_{i}=0)+(1-P_{0})(\frac{N_{i}}{Y_{i}})P{\left(x_{i}\right)}^{Y_{i}}{\left(1-P(x_{i})\right)}^{N_{i}-Y_{i}} \end{array}$$

To determine the best fit model for the nodule prevalence data, model fit statistics (DIC and WAIC) were calculated for each model, viz. binomial, ZIB Type I, and ZIB Type 0.

### Mesh construction

We assume an underlying spatially continuous variable for the observed geostatistical data, which can be modeled with Gaussian random fields. We used the Stochastic Partial Differential Equation (SPDE) approach in the *INLA* package to fit a spatial model and to predict each variable of interest at an unsampled location [2,52]. An approximate solution to SPDE can be found using the finite element method. The finite element representation of the Matérn field is used as a linear combination of basis functions defined on a triangulation of domain D [54]. Domain D is subdivided into a triangulated mesh which is formed first by placing the triangle’s vertices at the sample locations and then adding other vertices around the regions of spatial prediction.

We constructed the finite element mesh for SPDE approximation to the Gaussian process regression using the boundary of Ethiopia. Triangulation meshes with different cut-off parameters and the maximum length for the triangle inside and outside the boundary were tested for their model fit and computation cost. The mesh that yielded the lowest DIC and WAIC scores without significantly increasing computational cost was chosen.

### Cross-validation and prediction

K-fold cross-validation (with *k* = 10) was run to observe the differences in the predictive accuracy of the candidate models. Different measures of predictive accuracy were calculated by assessing the relationship between the predicted and observed prevalence in the validation dataset [39,46]. During each validation run, both Pearson correlation coefficient and the Root Mean Square Error (RMSE) between the observed data and the predicted data for validation samples were calculated to assess accuracy.

After assessing the accuracy of the candidate models, the best model was used for the prediction. The posterior distribution of prevalence was estimated at 5 km resolution, accounting for the effect of the variables and the spatial covariance structure. The covariate raster stack was aggregated to 5 km spatial resolution by taking either the mean or sum of the raster cells. The mean of raster cells was calculated for all continuous covariates except population count, for which the sum was calculated. Aggregated data were used to ease the computational burden associated with geospatial prediction at higher resolution. To visualize how uncertainty might impact whether a given pixel would be classified as hypoendemic based on the estimated nodule prevalence, we mapped exceedance probability using a threshold of 20%, i.e., the probability of the pixel being above the hypoendemic level. The map was also used to assess the relationship between the predicted prevalence and environmental variables using the *gam* smoothing function available in the *ggplot2* package in R [55] for visualization purposes.

We estimated the number of pre-intervention nodule positive people by multiplying the predicted posterior prevalence with the population count raster from the adjusted United Nations 2012 population counts derived from WorldPop [56]. We considered only the people living in rural areas, as done by O’Hanlon et al. [39], since onchocerciasis is primarily a disease of rural communities and is usually not found in urban areas [17,19]. Further, we calculated the aggregated mean cases, the range and standard deviation of predicted mean cases, and their uncertainty with respect to district/implementation units (IUs) using the estimated number of mean cases map and their lower and upper limit map.

## Results

We formulated a Bayesian geostatistical model using INLA to estimate the nationwide pre-intervention prevalence of *O*. *volvulus* nodules in Ethiopia. Nodule prevalence data from 916 unique geopositioned sites were combined with eight different environmental and socio-demographic covariates to construct the geostatistical model. Most of the prevalence data were from western Ethiopia, as eastern Ethiopia is largely unmapped for *O*. *volvulus* nodules prevalence. The mean and the standard deviation of the observed prevalence across the sampling locations in Ethiopia was 17.24±16.32% ranging from 0 to 81.48%. There were 204 sites with zero prevalence (Fig 1).

### Model selection and fitting

Four different types of models were tested for the nodule prevalence data, viz. binomial without spatial structure, binomial with spatial structure, ZIB type 1 and ZIB type 2, both with spatial structure. These were assessed without including any environmental and socio-demographic variables in the model. The binomial model that did not account for spatial effects showed higher DIC (9806.988) and WAIC (9816.581) scores (S4 Fig). The addition of spatial effects and accounting for zero inflation with a Type I zero-inflated binomial model decreased the DIC and WAIC scores to 5661.098 and 5916.715, respectively. In addition, nodule palpation is a low sensitivity diagnostic method, and the ZIB Type I model can account for possible false negatives. Thus, ZIB Type I with spatial structure was chosen for modeling the prevalence data and these results are presented in the main text. Note, however, that in the supplementary information we also provide the results for predicted mean prevalence and associated uncertainties obtained when using the regular binomial model with spatial effects (S5 Fig). There was no overall difference in the predicted prevalence map between the two models, but the magnitude of uncertainty in the predicted estimates was higher for the regular binomial model.

To optimize the SPDE mesh, six different triangulation meshes with different parameters were tested for their model fit and computation cost (S6 Fig, S4 Table). The mesh C yielded the best model fit scores (DIC = 4538.12; WAIC = 4652.22). However, the mesh E yielded a comparable model fit (DIC = 4572.74; WAIC = 4710.781) but was computationally more efficient (45.38 s vs. 1667.33 s) and, therefore, mesh E was chosen for fitting the model.

We selected environmental and socio-demographic variables based on the model fit scores of the univariate model. Isothermality was selected from the group of temperature variables, precipitation seasonality from the group of precipitation, and similarly, population density, distance to the nearest river, slope, and Normalized Difference Vegetation Index (NDVI) were selected from the group of socio-demographic, hydrological, and vegetation groups of covariates, respectively. Other combinations were also explored and the inclusion of covariates like soil moisture and flow accumulation further reduced the DIC and WAIC scores (S3 Table).

K-fold cross-validation (k = 10) was done for three different models: one without environmental covariates, one with six covariates, and the other with an additional two covariates (flow accumulation and soil moisture), which revealed that model 3 was superior to model 0 and 1 (S7 Fig). For model 3, calculating the Pearson correlation coefficient between the observed prevalence and the predicted prevalence ranged from 0.47 to 0.70 with a median of 0.65. Similarly, the RMSE ranged from 11.09 to 15.1, with a median of 13.18. This suggested a good model fit and accuracy for predictions across the validation datasets.

### Model parameters

The regression coefficients were estimated for each covariate included in the model. Since INLA is a Bayesian technique, the regression coefficients and their 95% Bayesian credible interval (BCI) are derived from a probability distribution rather than point estimates. We exponentiated the regression coefficients for each covariate to calculate the more interpretable risk ratio (RR). The RR for a covariate represents the ratio of nodule prevalence when the covariate is *x*+1 to the nodule prevalence when the covariate is *x*, holding all other variables constant [57]. The significance of the estimates was determined as described in Moraga et al. [58]. The association was deemed significant only if both the 95% BCI values were below 0 for negative association and above 0 for positive association. The RR is considered significant when the 95% BCI values do not overlap 1.

Out of 8 covariates considered for the final model, four covariates were significantly associated (based on 95% BCI) with *O*. *volvulus* nodule prevalence (Table 1). Soil moisture was significantly positively associated (RR: 1.0218, 95% BCI: 1.0135–1.0302) with *O*. *volvulus* nodule prevalence, whereas distance to the nearest river (RR: 0.9850, 95% BCI: 0.9751–0.995), precipitation seasonality (RR: 0.9837, 95% BCI: 0.9681–0.9995), and flow accumulation (RR: 0.9586, 95% BCI: 0.9321–0.9816) were negatively associated with *O*. *volvulus* nodules prevalence.

**Table 1:** Mean coefficient estimates and 95% Bayesian credible interval (BCI) for the environmental and socio-demographic variables in the model. Regression coefficients for covariates are presented as a risk ratio (RR), which represents the change in prevalence for a unit change in that covariate given that all other variables are kept constant. Significant covariates are followed by an asterisk (*).

	Regression coefficients	
**Variables**	**Risk ratio (RR)**	**95% BCI**
Distance to the nearest river	0.9850	(0.9751, 0.995)*
Soil moisture	1.0218	(1.0135, 1.0302)*
Flow accumulation	0.9586	(0.9321, 0.9816)*
Precipitation seasonality	0.9837	(0.9681, 0.9995)*
Vegetation index	1.0017	(0.9929, 1.0105)
Slope	1.0010	(0.9929, 1.009)
Population density	1.0000	(0.9997, 1.0003)
Isothermality	0.9982	(0.9717, 1.0252)
Intercept	0.1437	(0.0073, 2.8339)
**Hyper parameters**	**Mean coefficients**	**95% BCI**
Zero-inflation parameter	0.3334	(0.3275, 0.3422)
Range	3.1163	(2.9643, 3.3496)
Variance	1.4195	(1.1489, 1.8946)
95% BCI includes 0.025 quantiles and the 0.975 quantiles of the posterior probability distribution of the coefficients		

Hyperparameters defining the SPDE mesh were used to calculate the spatial effect and project the spatial field (S8 Fig). The spatial effect indicates the intrinsic spatial variability in the prevalence estimates, helping us understand the data’s spatial structure [47]. Further, the spatial field also represents the spatial effect that was not accounted for by the covariates included in the model [58]. The mean spatial field is higher in western Ethiopia while it is lower in central Ethiopia and eastern Ethiopia, along with the high standard deviation of the spatial field in the eastern parts. The zero-inflated parameter, which governs the probability of observing a zero prevalence, was reported to be 0.334 (95% BCI: 0.328–0.342), indicating the presence of significant zero inflation [48,59]. The spatial field showed a nominal range of 3.116 (95% BCI: 2.964–3.351) degrees which corresponds to the 345.38 (95% BCI: 329.00–375.81) km across the latitudinal range of Ethiopia, assuming 1 degree corresponds to 111 km for both latitude and longitude at the equator.

### Model prediction

**Fig 2:**
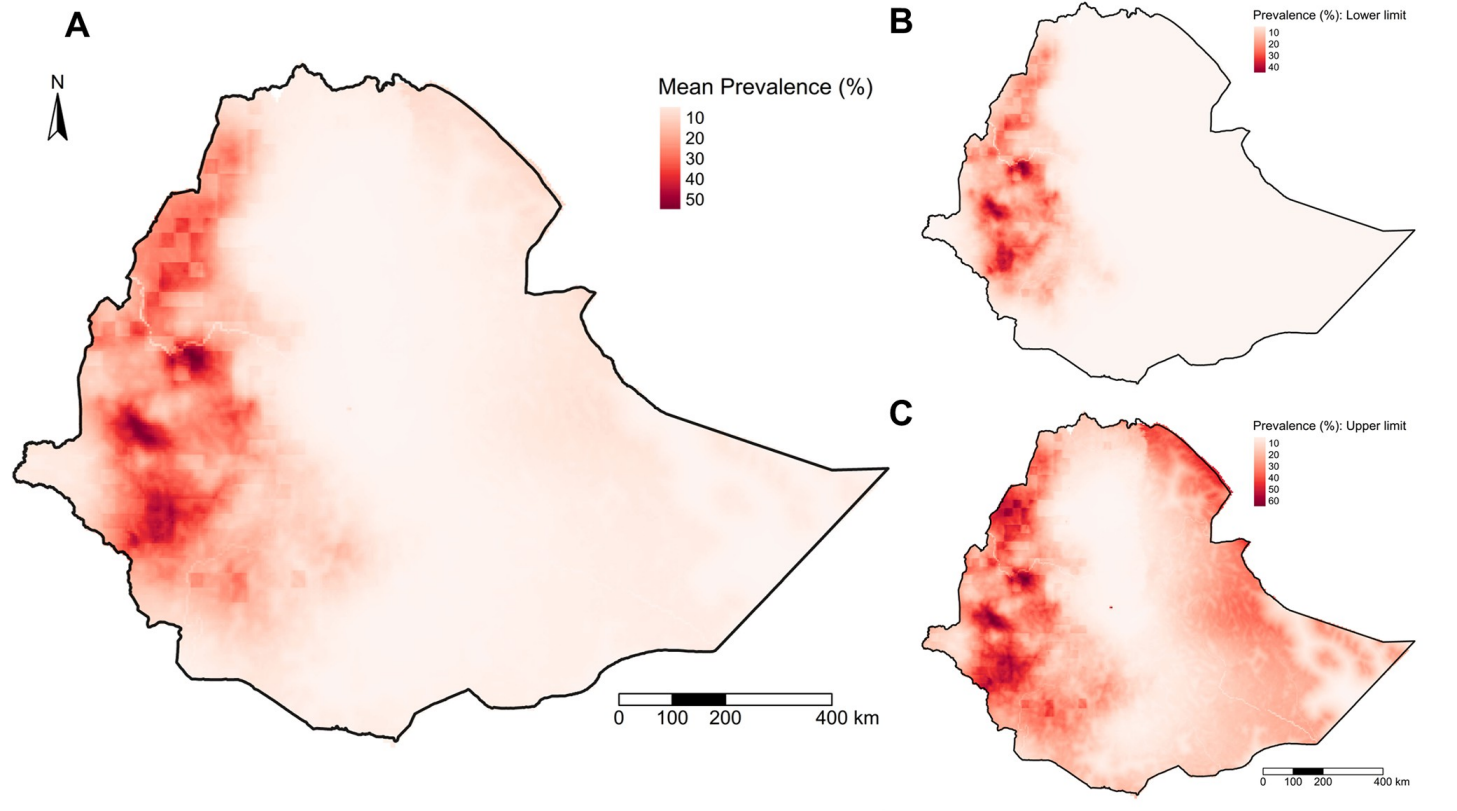
*Onchocerca volvulus* nodule posterior prevalence map in Ethiopia generated from the geostatistical model. (A) The mean, (B) the lower limit, and (C) the upper limit of *O*. *volvulus* nodule prevalence. The prediction interval of the prevalence map was generated from the calculated 95% BCI of fitted posterior prevalence values. The administrative borders are from the Global Administrative Areas (GADM) database (available at: https://gadm.org/maps.html).

**Fig 3:**
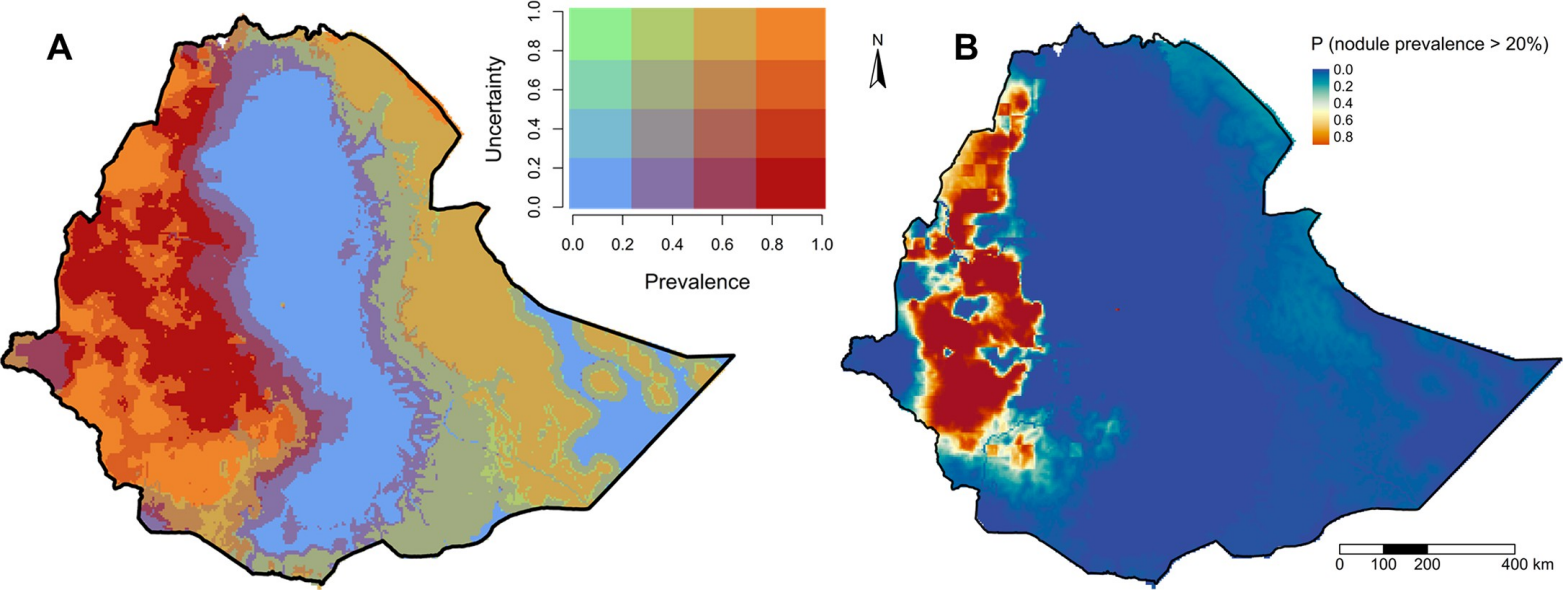
Uncertainty in the estimates of *O*. *volvulus* nodule prevalence from the model. (A) Exceedance threshold probability map that shows the posterior predictive probability that the nodule prevalence is greater than 20%. (B) Bivariate map that shows both prevalence and the uncertainty estimates rescaled from 0 to 1. The administrative borders are from the Global Administrative Areas (GADM) database (available at: https://gadm.org/maps.html).

The predicted prevalence map shows spatial heterogeneity in *O*. *volvulus* nodule prevalence in Ethiopia (Fig 2). Predicted *O*. *volvulus* nodule prevalence is concentrated in the western parts of Ethiopia, with three to four hotspots in southwest Ethiopia. There is a relatively low prevalence of nodules in eastern Ethiopia and near to zero prevalence in central Ethiopia. The range of predicted mean prevalence was 0.39 to 55.27%. Similarly, the lower limit of predicted nodule prevalence ranged from 0 to 47.28%, while the upper limit of the predicted prevalence ranged from 1.41 to 65.32%. The correlation between the observed and the predicted prevalence was 0.73 (S9 Fig). Due to the geostatistical smoothing effect, some observations with higher prevalence were underestimated and vice-versa. The uncertainty in the prevalence estimates was derived using the standard deviation of the posterior distribution and assessed using the 20% threshold exceedance probability map. The uncertainty map shows that the presence of data influenced the uncertainty in the prevalence estimates; i.e., areas with ground truth data have lower uncertainty (S5 Fig). The uncertainty was higher in eastern Ethiopia due to the lack of ground truth data from those sites. Most of central Ethiopia and some areas in eastern Ethiopia, regardless of the absence of the data, showed low prevalence with lower uncertainty (Fig 3A). There were areas with a high prevalence that had different levels of uncertainty in western Ethiopia. The regions with higher uncertainty almost always corresponded with sparse data from those regions. The exceedance probability map showed that there were a couple of clusters in west Ethiopia with high exceedance probability that the nodule prevalence exceeded 20% i.e., meso- or hyper- endemic (Fig 3B). The rest of Ethiopia has low exceedance probability of nodule prevalence > 20%. From the mean prevalence map, 126 thousand km^2^ of Ethiopia had predicted posterior mean nodule prevalence > 20%. However, using a threshold probability of 0.9, only 6.94% of the total area of Ethiopia exceeded 20% prevalence, corresponding to around 77 thousand km^2^ and a 2012 population of 4.86 million. Using an exceedance probability of 0.5, 17.5% of the total area of Ethiopia exceeds 20% nodule prevalence, which is equivalent to 193.25 thousand km^2^.

**Fig 4:**
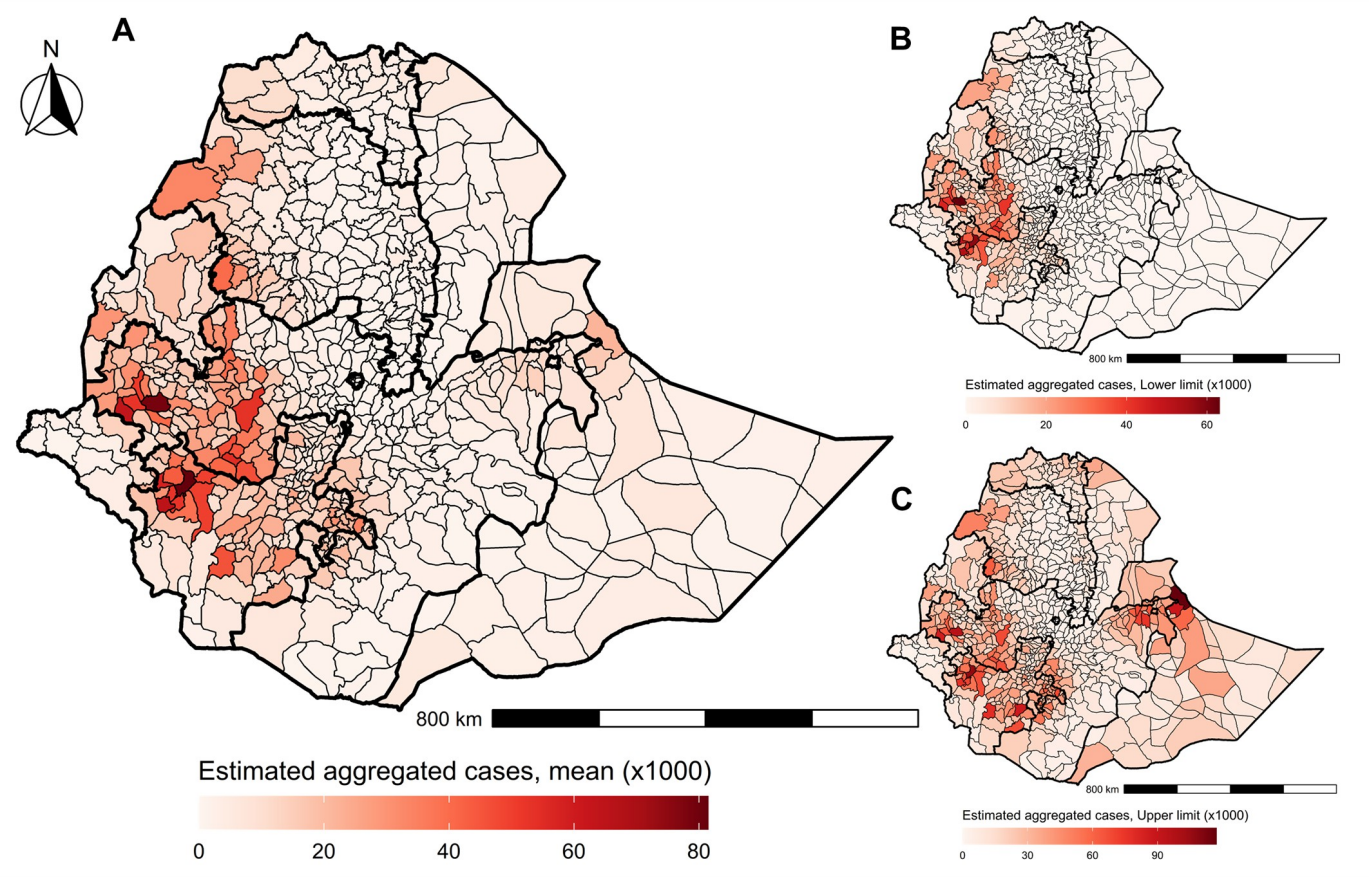
The estimated number of pre-intervention *O*. *volvulus* nodule prevalence cases with respect to Ethiopian districts. (A) The mean pre-intervention cases, (B) their upper and (C) lower limit were aggregated at the district level border. The regional borders are highlighted with thicker lines. The administrative borders are from the Global Administrative Areas (GADM) database (available at: https://gadm.org/maps.html).

We estimated the number of pre-intervention cases to be 6.48 million (95% BCI: 3.53–13.04 million), which was 9.08% (95% BCI: 5.12%–17.26%) of the total rural population of Ethiopia in 2012. We aggregated the pixel-level case numbers to the district level to create an aggregated case number map with lower and upper limits (Fig 4). The model estimated that the woredas/districts within Southern Nations, Nationalities and Peoples (SNNPR), and Oromia region had the highest number of pre-intervention onchocerciasis nodule cases (predicted mean cases in the range of 50–80 thousand) prior to intervention. As expected, some districts within the Addis Abeba region were predicted to have nil cases. Similarly, a district-level map was created by aggregating the mean prevalence from pixels within the respective districts which represent implementation units (IUs) for MDAi (S10 Fig). The aggregated mean prevalence for the first dozen of the most endemic districts was greater than 40%. The difference between the highest and lowest estimated prevalence pixels (range of mean prevalence) within the districts was as high as 50.72% for a district within the Kemashi zone of Ethiopia.

### Relationship of environmental and socio-demographic covariates on the prevalence

**Fig 5:**
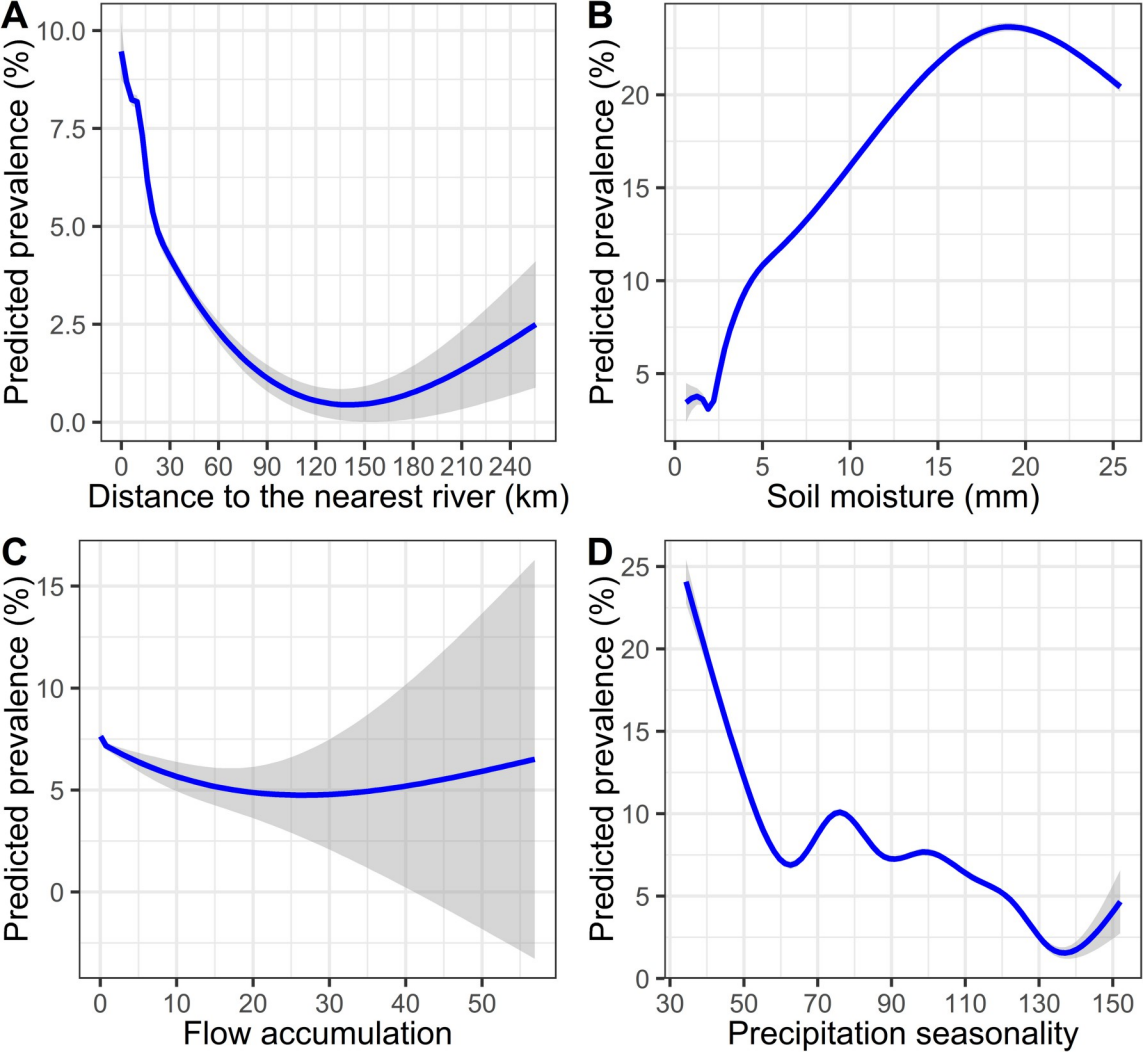
The relationship between the predicted posterior mean prevalence with the significant environmental covariates. The curve was fitted using the *gam* smoothing function available in the *ggplot2* package for the purpose of visualization. The shaded region around each curve represents the 95% confidence interval. Flow accumulation had a range of high magnitude compared to other covariates (values ranged from 0 to 100418). Thus, this variable was rescaled from 0 to 100 to make its range comparable with other variables.

A smoothing curve was fitted between the predicted posterior mean of the nodule prevalence from the generalized linear model and the covariates used in the model to assess the relationship between them. The relationship profile of the predicted *O*. *volvulus* nodule prevalence across the range of values of different covariates indicates which ecological conditions are suitable for onchocerciasis transmission (Fig 5). The relationship curve for the distance to the nearest river and the predicted prevalence shows a sharp decline in the *O*. *volvulus* nodule prevalence to around 20–25 km, and the curve continues in the low prevalence region with increased uncertainty as distance increases from the nearest river (Fig 5). There was almost a linear increase in the predicted prevalence with an increase in soil moisture up to around 18 mm.

There was a negative association with flow accumulation with a considerable increase in uncertainty in the areas with high flow accumulation, i.e., larger rivers. Nevertheless, the areas with lower flow accumulation had a higher predicted prevalence than those with higher flow accumulation, suggesting the importance of intermediate-sized rivers to onchocerciasis epidemiology in Ethiopia. In addition, the relationship curve for the slope shows that a certain degree of slope is favorable for *O*. *volvulus* nodule prevalence (S11 Fig). There is a similar response profile for population density where intermediate population density is favorable for onchocerciasis transmission. There was a steep decline in predicted prevalence with the initial increase in precipitation seasonality. However, there was a mixed non-linear relationship in the regions with precipitation seasonality from 60 to 130 mm.

## Discussion

We generated a country-level geospatial map of *O*. *volvulus* nodule prevalence before the start of MDAi in Ethiopia, accounting for environmental and socio-demographic factors. The prevalence has been extrapolated to the country-level border of Ethiopia, including the eastern regions which were not mapped previously. Predicted prevalence in areas where people do not currently inhabit can indicate the risk of transmission should infected people establish communities. Prevalence was estimated using the pre-intervention nodule prevalence data and therefore represents the onchocerciasis status before MDAi in Ethiopia. Thus, these predictions can act as a pre-control baseline map to prioritize mapping of likely hypoendemic areas that are not yet under MDAi and to assess the effects of past interventions or of ecological changes at different locations.

The predicted nodule prevalence was found to be relatively low in the central parts of Ethiopia. This can be attributed to the presence of a significant geographical feature: the Great Rift Valley system. The elevated highlands along the center and lowland to the east of the Great Rift Valley are characterized by low predicted prevalence. The land east of the valley is dry with few rivers [16,20]. On the other hand, the combination of high elevation, steep slopes, and abundant rainfall in western Ethiopia result in fast-flowing rivers, a specific requirement for blackfly breeding and development.

The spatial pattern of *O*. *volvulus* nodule prevalence predicted across Ethiopia by this model was consistent with previously published prevalence maps that were based on REMO and other data [17,19]. Zouré et al. [17], using the same REMO data that we use here, built their model using data across sub-Sarahan Africa, although only included western Ethiopia and did not incorporate ecological characteristics. They predicted a similar area with positive nodule prevalence (126 thousand km^2^ vs 124.5 thousand km^2^). However, the total number of nodule-positive people across Ethiopia (2.88 million, 95% quantile interval: 2.68–3.12 million) was predicted to be considerably lower compared to our model results (6.48 million, 95% BCI: 3.53–13.04 million). The areas showing 20% threshold exceedance probability were analogous.

In previously published maps and in this predicted map, there was a high level of spatial heterogeneity in nodule prevalence, including heterogeneity within health districts (which are the implementation units for MDAi in Ethiopia). The difference between the highest and lowest prevalence pixel within the districts was as high as 50% (S10 Fig). A study in Cameroon reported that hypoendemic areas could sustain low-grade transmission and, therefore, might cause rapid recrudescence in neighboring meso- and hyperendemic areas where transmission has been successfully controlled [60]. Given that many of the unmapped onchocerciasis-endemic areas of Ethiopia are hypoendemic, these areas must be identified and treated to reach elimination of transmission. Hence, we need to consider the spatial heterogeneity within and between the intervention units when programs plan for elimination.

We used a bivariate map to visualize the estimated mean prevalence and associated uncertainty (Fig 3). The presence/absence of data influences this uncertainty map; i.e., areas with ground truth data have lower prediction errors because the prediction power decreases as the distance increases from a data point [39]. Thus, the uncertainty map can indicate where additional data would reduce the overall prediction error of the prevalence map, particularly in areas with higher prevalence and identify regions that might benefit from targeted re-mapping or elimination mapping efforts [3]. For example, there are areas with higher prevalence in the west but varying uncertainty that would be suitable for re-mapping. Similarly, there are areas in the east with both low prevalence and lower uncertainty, i.e., with higher confidence, and thus, do not need to be re-mapped.

### Ecological features associated with *O*. *volvulus* nodule prevalence

The major environmental factors significantly associated with nodule prevalence were distance to the nearest river, soil moisture, precipitation seasonality, and flow accumulation. As expected, there was a negative association between the distance to the nearest river and predicted prevalence. Onchocerciasis has long been recognized as being higher in communities near rivers and this correlation, which has been reported in prior geospatial modeling studies [19,37,39], is driven by blackfly breeding and development requirements for fast-flowing rivers, such that villages can be categorized epidemiologically as first, second, or third-line villages based on their proximity to vector breeding sites [39,61,62].

The relationship curve between the predicted prevalence and the distance to the nearest river shows that there is an initial rapid decline in prevalence followed by a less rapid decline as the distance from the river increases, and the curve asymptotes to a very low prevalence with increased uncertainty as the distance exceed 100 km. A rapid decline in blackfly biting rate at increasing distance from a river breeding site, based on vector biting rate data collected in northern Cameroon over three years, has been reported previously [63]. Similarly, a mark-recapture study found a logarithmic decline in the proportional fly biting density as the distance increased from the marking site [64], and a mark-release-recapture study in Ghana in West Africa reported the average flight range of *S*. *damnosum* may be as high as 27 km [62]. While Ethiopia is host to several different competent blackfly vector species, the part of the curve where the change in slope declines is consistent with this estimated flight range viz., ~20–25 km. However, the curve does not reach its lowest point until 100 km, suggesting that the parasites could be transmitted beyond the average dispersal range of an individual blackfly. This could be because the dispersal range for gravid blood-seeking and ovipositing female blackflies has been reported to be greater than the average dispersal range at around 60–100 km from the river [62]. In addition, wind-assisted long-distance migration of blackflies of hundreds of km and transmission due to the human migration have also been reported [65–67]. Thus, this study supports that longer range migration is likely and contributes to *O*. *volvulus* transmission.

We observed a positive association between soil moisture and *O*. *volvulus* nodule prevalence. Soil moisture is high in areas with high precipitation or near water bodies, including rivers—i.e., where there are suitable blackfly breeding sites. Soil moisture is an indicator of agricultural suitability, and agricultural areas have historically been known to have a high prevalence of onchocerciasis [19,37,68]. Agricultural lands and farms in these areas tend to be near rivers for easy irrigation. Therefore, the increased prevalence of *O*. *volvulus* nodules among people involved in agriculture and farming [37,69,70] is presumably because these workers are generally outdoors, often in proximity to rivers, and thus experience increased exposure to blackflies [7,9,71].

Flow accumulation is used in hydrogeology as a proxy for river grades and represents the cumulative number of cells in a raster object that flow into a given cell: high flow accumulation represents large rivers, and low flow accumulation represents secondary rivers and their tributaries. It has been used to map onchocerciasis hotspots in hypoendemic settings of the Democratic Republic of Congo [72]. In this study, flow accumulation was negatively associated with *O*. *volvulus* nodule prevalence, meaning that onchocerciasis was more common in communities near secondary rivers and tributaries than those near large rivers. Rivers with high flow accumulation are less likely to have the white water rapids optimal for *Simulium* breeding and development [73]. In Ethiopia, the primary vectors of onchocerciasis are *S*. *damnosum s*.*l*. and *S*. *neavei* [74]. *Simulium damnosum* in west Africa are more common in rivers of medium width (lower flow accumulation) than in large size rivers [6]. *Simulium neavei* has an obligatory phoretic association of larvae with freshwater crabs that are more common in sheltered, small forest streams [75,76] that form a dominant ecotype in southwestern Ethiopia where most of the data were collected for this study [9,77].

Precipitation seasonality, which refers to the variation in precipitation over a year, was also negatively associated with the predicted prevalence; i.e., the prevalence was high in areas with lower precipitation seasonality. Precipitation seasonality is calculated as a ratio of the standard deviation of the monthly total precipitation to the mean of the monthly precipitation expressed as percentage [78]. Areas with high precipitation seasonality might have ephemeral rather than perennial streams. If breeding sites are ephemeral, blood-feeding by blackflies would only happen during some parts of the year, lowering the annual biting rate, a key parameter in *O*. *volvulus* transmission [79–81]. Southeast Ethiopia, characterized by low prevalence of *O*. *volvulus* nodules, has high seasonality in precipitation characterized by two short wet seasons with a dry period in between [82]. However, the southwestern areas where the disease is most endemic have low precipitation seasonality and high annual precipitation [83].

As one would expect, the environmental factors significantly associated with *O*. *volvulus* nodule prevalence all exert a strong influence on vector breeding and thus blackfly density and biting rates. This strong association between determinants of vector breeding and nodule prevalence implies that spatial variation in vector breeding drives the spatial variation of *O*. *volvulus* nodule prevalence in Ethiopia and that the geospatial model we present here, based on nodule prevalence data, is also predictive of vector distribution. Changes in precipitation patterns due to ongoing climate change [84,85] and other anthropogenic environmental impacts such as irrigation or the construction of hydroelectric dams might significantly change vector distribution and thus the spatial occurrence of the disease [73,86]. The impacts of these changes could be modeled using the approach introduced here [87,88].

### Model limitations and recommendations

The geospatial model we report here incorporates different environmental and socio-demographic variables that are known to influence the transmission and prevalence of *O*. *volvulus* and the distribution of blackflies. However, the data incorporated in the model do not include all factors that may be epidemiologically relevant, such as direct/indirect interventions affecting onchocerciasis prevalence and human behaviors that may increase or decrease the risk of infection. The non-uniform mean spatial field across the triangulation mesh shows that there might be some effects that are unaccounted for by the model (S8 Fig), and the possibility remains that an unidentified covariate that closely resembles the spatial field might aid in explaining the spatial variation in prevalence. In addition, the inclusion of blackfly distribution maps based on the identification of breeding sites and their productivity might improve the model fit. Unfortunately, such data are not available for Ethiopia.

Some variables that we expected to correlate with prevalence, such as human host population density and vegetation, were not shown to be significantly associated in these analyses. Blackflies are not usually reported in dense urban environments while vegetation cover is essential for blackfly breeding [37, 68, 89]. We suggest that the lack of association might be because the country-wide spatial scale neutralizes factors that impact prevalence at a smaller geographic scale. Therefore, targeted spatial analysis in regions with differences in vegetation near rivers or with differences in rural-urban indices [90] might be helpful to explore the effects of these variables on nodule prevalence. Furthermore, *O*. *volvulus* transmission is highly dynamic not just spatially but also temporally. Extending the current spatial model to a spatio-temporal model might improve the model fit, but would require prevalence data at a temporal resolution that is not currently available for Ethiopia.

While the specificity of nodule palpation as a diagnostic technique for onchocerciasis is high, it is not sensitive [14,18,91,92]. Therefore, onchocerciasis infection based on nodule prevalence may be underestimated pre-MDAi. We could not include prevalence measures based on other diagnostic methods because fine-scaled prevalence data based on mf counts from skin snips or antibody tests (Ov16) were not available for Ethiopia. However, these data could be used as an alternative to, or in addition to, nodule prevalence. Combining data across methods is challenging, as correlations between mf prevalence and nodule prevalence can be highly variable (S1 Fig; however, see [18]) and the correlation between either of these measures and Ov16 seropositivity prevalence is unclear. Nevertheless, the map presented here could be used by onchocerciasis elimination programs to direct resources for elimination mapping because elimination mapping of any disease can be expensive [3], and the method described here may be an inexpensive first step that can extrapolate country-wide prevalence from existing data and thus better target re-mapping efforts.

## Conclusion

Onchocerciasis programs have transitioned from control of onchocerciasis as a public health problem to elimination of *O*. *volvulus* transmission, triggering the need to develop new tools to more efficiently prioritize decisions concerning elimination mapping and interventions in hypoendemic foci that were not previously targeted for intervention. To this end, we have generated a baseline pre-intervention prevalence map for the whole of Ethiopia using geospatial modelling based on pre-intervention nodule prevalence data and spatial variation in different environmental and socio-demographic factors. We extrapolated existing historical nodule prevalence measures to previously unmapped regions of Ethiopia and quantified uncertainty in predicted prevalence. This map could be used as an aid to decision making on where and how to (a) extend elimination mapping into areas identified as likely hypoendemic foci and (b) prioritize the allocation of scarce health system resources to areas most likely to benefit from that allocation. Furthermore, this study found that hydrological variables such as distance to the nearest river, soil moisture, precipitation seasonality, and flow accumulation were significant in describing the spatial heterogeneity of *O*. *volvulus* nodules in Ethiopia. All these ecological features are related to the suitability of an area for vector breeding, movement, biting behavior, and density, leading to the conclusion that vector suitability and movement are the primary determinants of the spatial distribution of *O*. *volvulus* nodules in Ethiopia. Consequently, changes in these ecological features due to anthropomorphic changes in climate, agriculture, vegetation type (e.g., slash-and-clear), or construction of hydroelectric or irrigation dams might significantly alter the occurrence of the disease. We suggest, therefore, that the importance of these vector-related ecological factors in determining onchocerciasis distribution and intensity reaffirms that inclusion of vector control could augment current interventions based primarily on prophylactic chemotherapy.

## Supporting information

S1 Table Information on environmental and socio-demographic covariates considered for the geospatial analysis. (XLSX)

S2 Table Variable inflation factor (VIF) for 15 covariates selected during the initial round of variable selection. (XLSX)

S3 Table Information criterion scores for each potential explanatory variable(s). A univariate spatial model was fitted to each variable, and the DIC and WAIC scores were calculated. Variables were grouped into the category they represented, and the variable with the least DIC and WAIC scores were selected. Other variables were explored after combining the selected variables.
(XLSX)

S4 Table The parameters, model fit scores, and the computational cost for different meshes shown on S4 Fig. A reasonable improvement in model fit was achieved with Mesh E without compromising the computational cost. Thus, Mesh E was chosen to represent the Matérn field. The time taken for the computation is based on a machine with Intel i7, 3.8 GHz processor.
(XLSX)

S1 Fig Correlation between nodule prevalence and microfilarial prevalence data from the identical geo-locations. The Pearson correlation coefficient and significance estimated from 44 sites are shown on the top left, indicating poor correlation.
(DOCX)

S2 Fig Socio-demographic and environmental covariates used in the geostatistical model. The raster layers are masked to the border of Ethiopia. Flow accumulation and NDVI are rescaled from 0 to 100. NDVI: Normalized Difference Vegetation Index. The administrative borders are from the Global Administrative Areas (GADM) database (available at: https://gadm.org/maps.html).
(DOCX)

S3 Fig Correlation matrix of the 15 environmental and socio-demographic variables selected after the initial round of covariate selection. Spearman’s rank correlation coefficient was estimated assuming the non-normality of the data, and the correlation coefficient for each pair of covariates was below 0.8. ELV: elevation; ADR: annual diurnal range; IST: isothermality; PWTQ: precipitation wettest quarter; PST: precipitation seasonality; PWMQ: precipitation warmest quarter; PCQ: precipitation coldest quarter; NDVI: normalized difference vegetation indices; DTR: Distance to the nearest river; FAC: flow accumulation; SLP: slope; SLM: soil moisture; PDT: population density; NLT: night lights; PHI: prevalence of housing improvement.
(DOCX)

S4 Fig Changes in the model fit statistics for different types of models. Type 1 zero-inflated binomial distribution yielded the lowest AIC and WAIC scores suggesting the best model fit.
(DOCX)

S5 Fig Predicted output from the regular binomial model. Predicted mean (**A**) of the posterior prevalence and their upper (**B**) and the lower limit (**C**) calculated based on 95% BCI. The 20% threshold exceedance probability map (**D**) is also shown for the binomial model and also the uncertainty measured as the standard deviation of the predicted posterior prevalence for the binomial (**E**) and the Type I zero inflated binomial model (**F**). The data location is indicated by ‘+’ on the map showing uncertainty influenced by presence of the data. The magnitude of uncertainty is higher for the regular binomial model compared to the Type I zero inflated binomial model. The administrative borders are from the Global Administrative Areas (GADM) database (available at: https://gadm.org/maps.html).
(DOCX)

S6 Fig Different triangulated SPDE meshes considered for the analysis. The country boundary for the prediction region and the location of the observations (blue points) are shown on the triangulation mesh. The finer mesh is within the country boundary, while the coarser mesh is present outside the country boundary in the buffer region. The administrative borders are from the Global Administrative Areas (GADM) database (available at: https://gadm.org/maps.html).
(DOCX)

S7 Fig Boxplot showing cross-validation statistics obtained from 10-fold cross-validation from the three different geostatistical models. (A) Pearson correlation coefficient and (B) root mean square error (RMSE) were calculated between the predicted and the observed prevalence of each validation set during each cross-validation run. Model 0 is the model with only intercept and the spatial field, model 1 consists of six variables (slope, isothermality, precipitation seasonality, normalized difference vegetation index (NDVI), population density, and distance to the nearest river) with intercept and the spatial field, and model 2 consists of everything in model 1 with two additional variables (flow accumulation and soil moisture).
(DOCX)

S8 Fig The spatial field’s posterior mean (A) and standard deviation (B) from the stochastic partial differential equation (SPDE) mesh. The spatial field is higher in western Ethiopia. While the spatial field is lower in eastern Ethiopia, the standard deviation of the spatial field is higher. The administrative borders are from the Global Administrative Areas (GADM) database (available at: https://gadm.org/maps.html).
(DOCX)

S9 Fig Correlation between the observed and predicted prevalence. The dashed line is the expectation for perfect correlation. The Pearson correlation coefficient and associated p-value are shown on the bottom left of the plot. Points are colored by the absolute difference between the observed and the predicted prevalence.
(DOCX)

S10 Fig The aggregated mean prevalence and range of the estimated mean prevalence within Ethiopian districts. (A) The mean of the estimated prevalence of all the pixels within the district level border and (B) the range of the estimated prevalence within the district, i.e. the difference between the highest prevalence pixel and the lowest prevalence pixel. The administrative borders are from the Global Administrative Areas (GADM) database (available at: https://gadm.org/maps.html).
(DOCX)

S11 Fig The relationship between the predicted posterior mean prevalence and the non-significant environmental and socio-demographic covariates in the regression model. The curve was fitted using the *gam* smoothing function available in the *ggplot2* package for the purpose of visualization. The shaded region around the curve represents the 95% confidence interval. NDVI was rescaled from 0 to 100. NDVI: Normalized Difference Vegetation Index.
(DOCX)
