# Supplementary material for: Geospatial modeling of pre-intervention nodule prevalence of Onchocerca volvulus in Ethiopia as an aid to onchocerciasis elimination
Source: PLoS Negl Trop Dis. 2022 Jul 18;16(7):e0010620. doi: 10.1371/journal.pntd.0010620 (PMC9333447; doi:10.1371/journal.pntd.0010620)
Supplement: S1 Fig — The Pearson correlation coefficient and significance estimated from 44 sites are shown on the top left, indicating poor correlation. (DOCX) [file pntd.0010620.s005.docx]

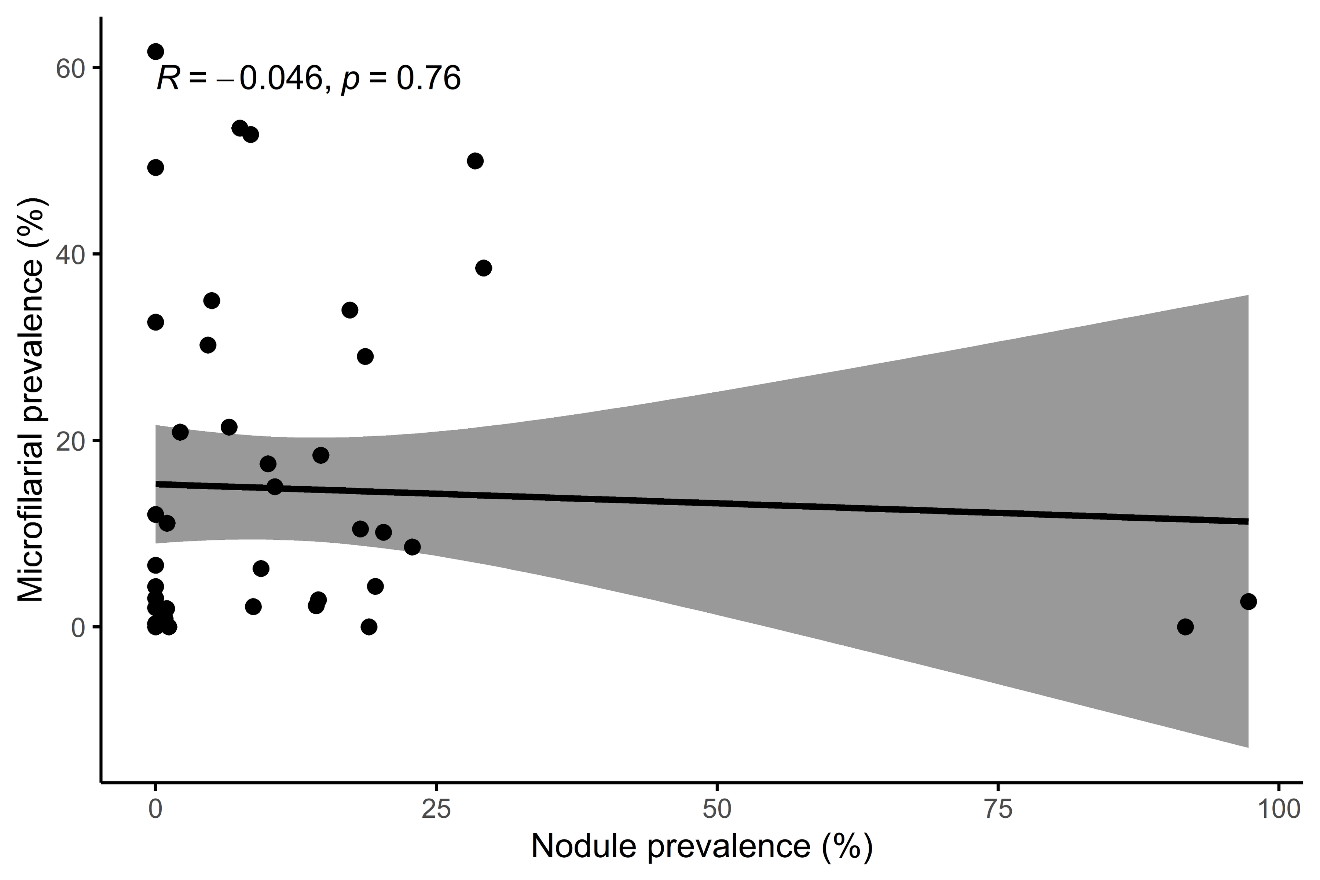


**S1 Fig. Correlation between nodule prevalence and microfilarial prevalence data from the identical geo-locations.** The Pearson correlation coefficient and significance estimated from 44 sites are shown on the top left, indicating poor correlation.
