# Supplementary material for: Geospatial modeling of pre-intervention nodule prevalence of Onchocerca volvulus in Ethiopia as an aid to onchocerciasis elimination
Source: PLoS Negl Trop Dis. 2022 Jul 18;16(7):e0010620. doi: 10.1371/journal.pntd.0010620 (PMC9333447; doi:10.1371/journal.pntd.0010620)
Supplement: S2 Fig — The raster layers are masked to the border of Ethiopia. Flow accumulation and NDVI are rescaled from 0 to 100. NDVI: Normalized Difference Vegetation Index. The administrative borders are from the Global Administrative Areas (GADM) database (available at: https://gadm.org/maps.html). (DOCX) [file pntd.0010620.s006.docx]

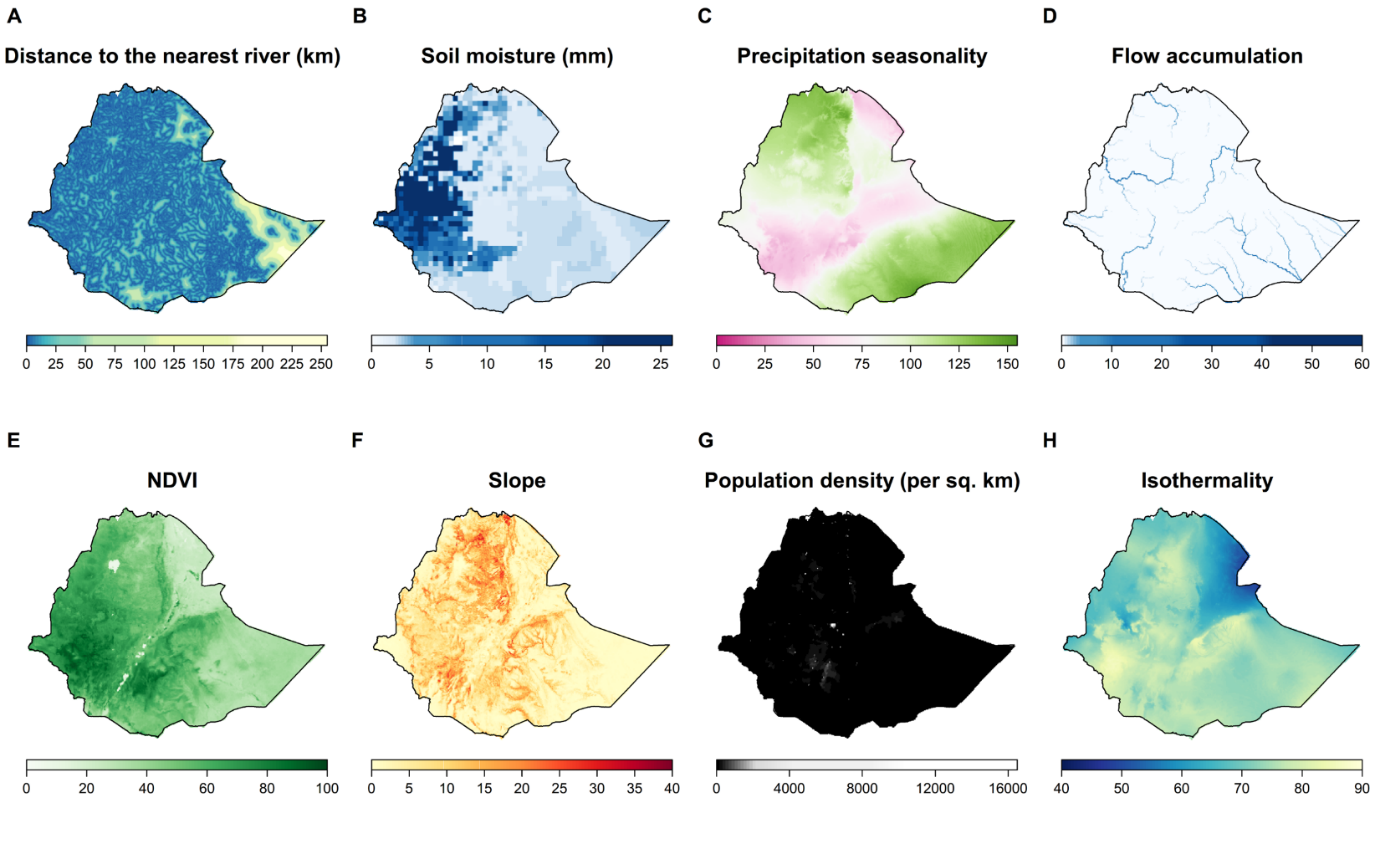


**S2 Fig. Socio-demographic and environmental covariates used in the geostatistical model.** The raster layers are masked to the border of Ethiopia. Flow accumulation and NDVI are rescaled from 0 to 100. NDVI: Normalized Difference Vegetation Index. The administrative borders are from the Global Administrative Areas (GADM) database (available at: https://gadm.org/maps.html).
