# Supplementary material for: Geospatial modeling of pre-intervention nodule prevalence of Onchocerca volvulus in Ethiopia as an aid to onchocerciasis elimination
Source: PLoS Negl Trop Dis. 2022 Jul 18;16(7):e0010620. doi: 10.1371/journal.pntd.0010620 (PMC9333447; doi:10.1371/journal.pntd.0010620)
Supplement: S3 Fig — Spearman’s rank correlation coefficient was estimated assuming the non-normality of the data, and the correlation coefficient for each pair of covariates was below 0.8. ELV: elevation; ADR: annual diurnal range; IST: isothermality; PWTQ: precipitation wettest quarter; PST: precipitation seasonality; PWMQ: precipitation warmest quarter; PCQ: precipitation coldest quarter; NDVI: normalized difference vegetation indices; DTR: Distance to the nearest river; FAC: flow accumulation; SLP: slope; SLM: soil moisture; PDT: population density; NLT: night lights; PHI: prevalence of housing improvement. (DOCX) [file pntd.0010620.s007.docx]

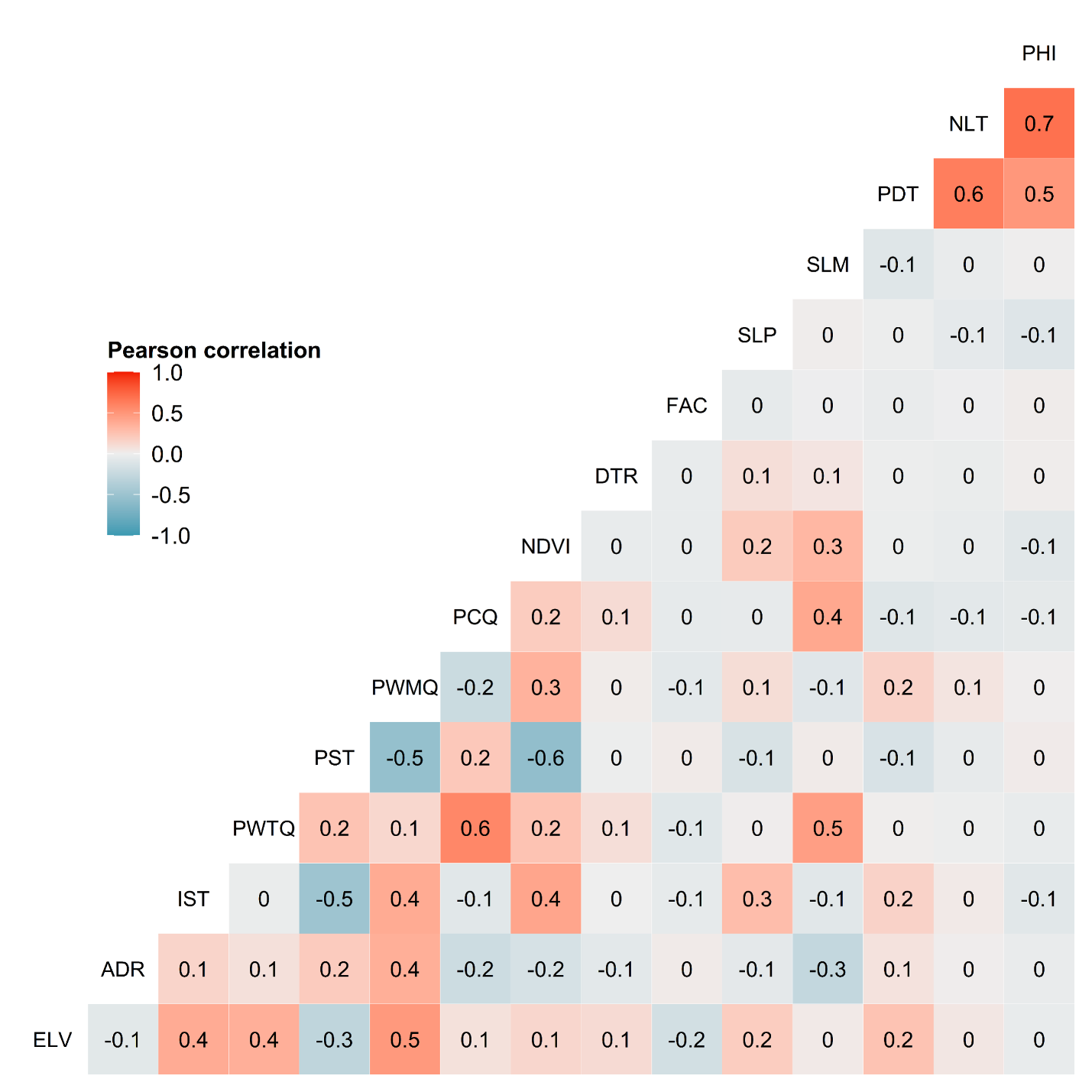


**S3 Fig. Correlation matrix of the 15 environmental and socio-demographic variables selected after the initial round of covariate selection.** Spearman's rank correlation coefficient was estimated assuming the non-normality of the data, and the correlation coefficient for each pair of covariates was below 0.8. ELV: elevation; ADR: annual diurnal range; IST: isothermality; PWTQ: precipitation wettest quarter; PST: precipitation seasonality; PWMQ: precipitation warmest quarter; PCQ: precipitation coldest quarter; NDVI: normalized difference vegetation indices; DTR: Distance to the nearest river; FAC: flow accumulation; SLP: slope; SLM: soil moisture; PDT: population density; NLT: night lights; PHI: prevalence of housing improvement.
