# Supplementary material for: Geospatial modeling of pre-intervention nodule prevalence of Onchocerca volvulus in Ethiopia as an aid to onchocerciasis elimination
Source: PLoS Negl Trop Dis. 2022 Jul 18;16(7):e0010620. doi: 10.1371/journal.pntd.0010620 (PMC9333447; doi:10.1371/journal.pntd.0010620)
Supplement: S4 Fig — Type 1 zero-inflated binomial distribution yielded the lowest AIC and WAIC scores suggesting the best model fit. (DOCX) [file pntd.0010620.s008.docx]

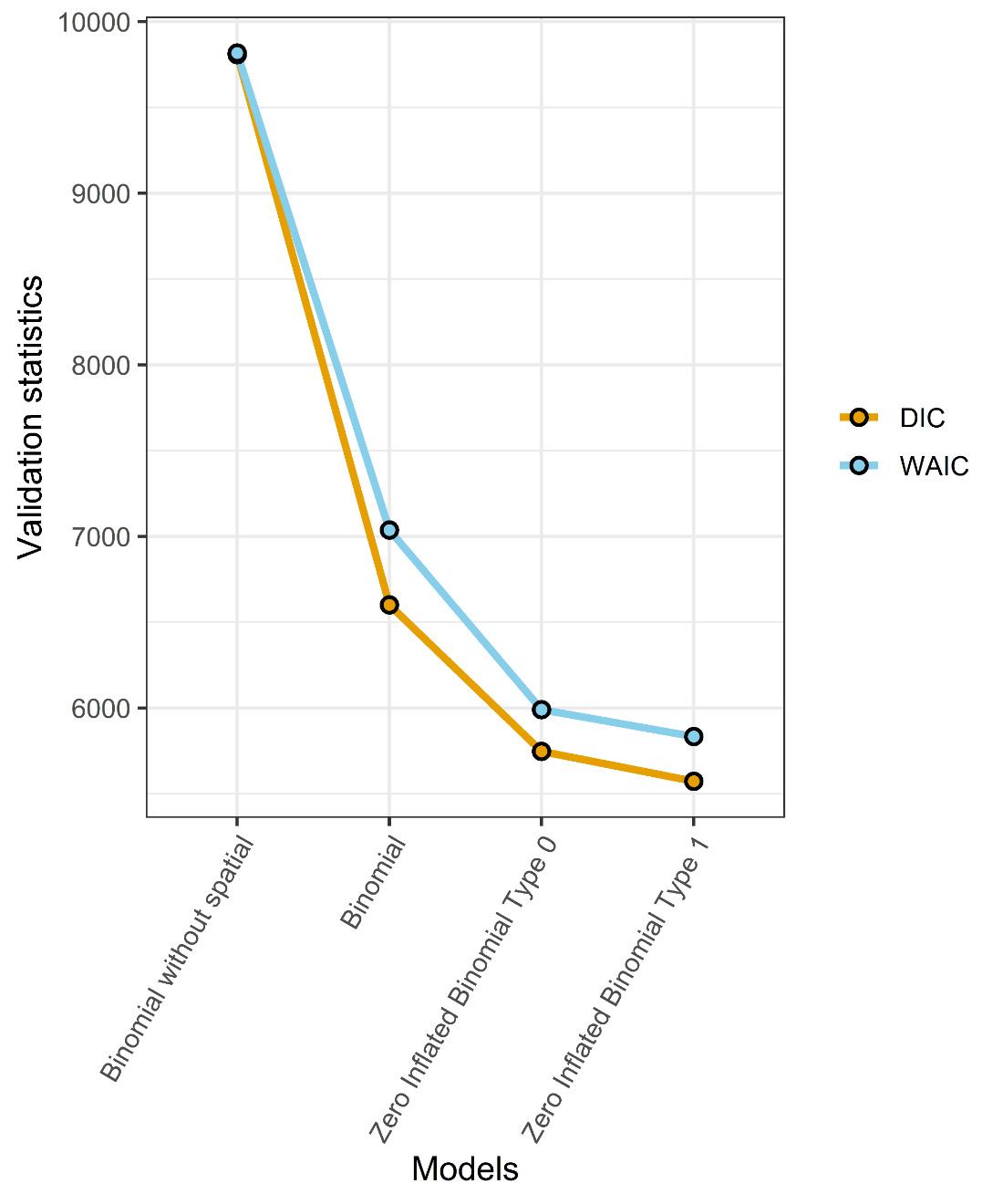


**S4 Fig. Changes in the model fit statistics for different types of models.** Type 1 zero-inflated binomial distribution yielded the lowest AIC and WAIC scores suggesting the best model fit.
