# Supplementary material for: Geospatial modeling of pre-intervention nodule prevalence of Onchocerca volvulus in Ethiopia as an aid to onchocerciasis elimination
Source: PLoS Negl Trop Dis. 2022 Jul 18;16(7):e0010620. doi: 10.1371/journal.pntd.0010620 (PMC9333447; doi:10.1371/journal.pntd.0010620)
Supplement: S5 Fig — Predicted mean (A) of the posterior prevalence and their upper (B) and the lower limit (C) calculated based on 95% BCI. The 20% threshold exceedance probability map (D) is also shown for the binomial model and also the uncertainty measured as the standard deviation of the predicted posterior prevalence for the binomial (E) and the Type I zero inflated binomial model (F). The data location is indicated by ‘+’ on the map showing uncertainty influenced by presence of the data. The magnitude of uncertainty is higher for the regular binomial model compared to the Type I zero inflated binomial model. The administrative borders are from the Global Administrative Areas (GADM) database (available at: https://gadm.org/maps.html). (DOCX) [file pntd.0010620.s009.docx]

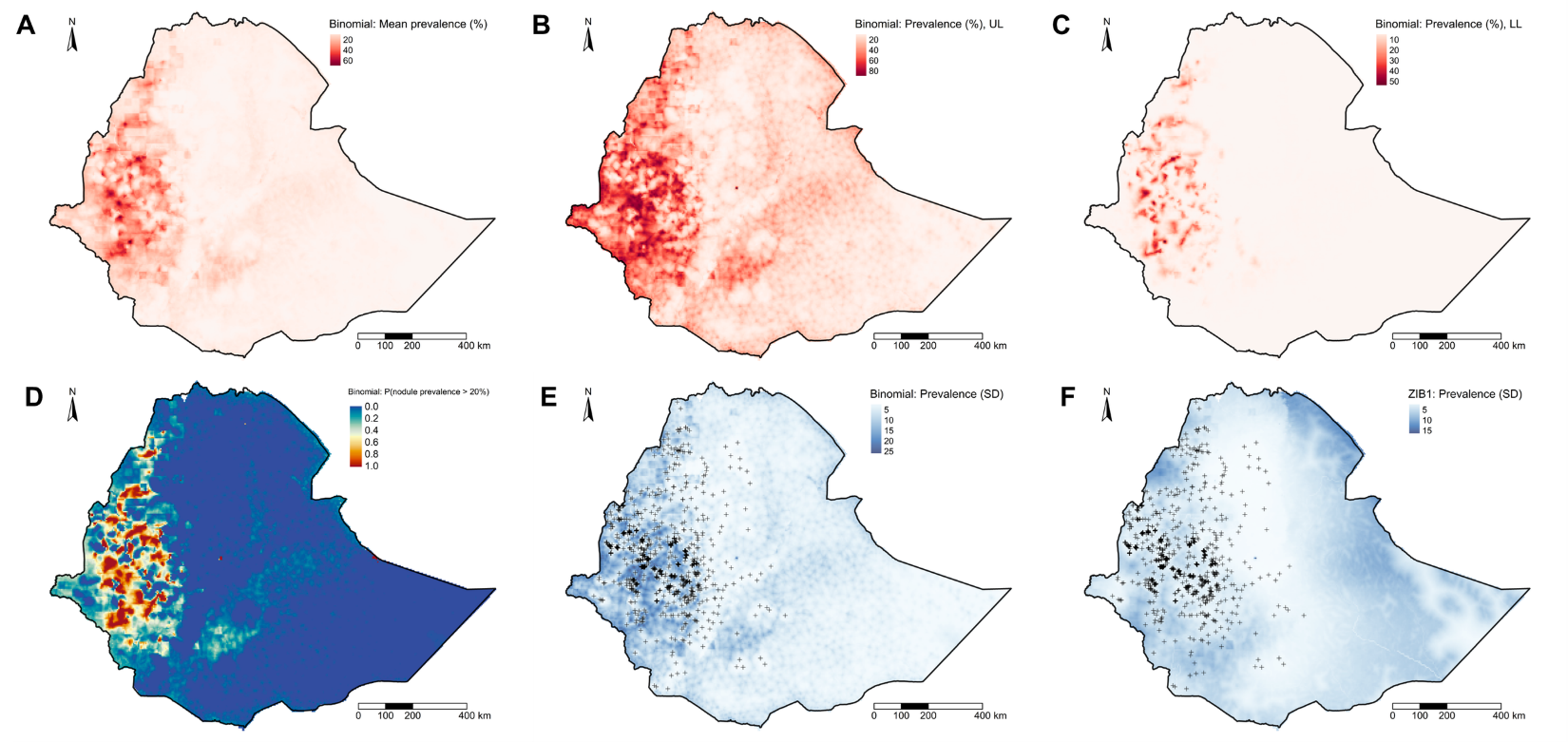


**S5 Fig. Predicted output from the regular binomial model.** Predicted mean (**A**) of the posterior prevalence and their upper (**B**) and the lower limit (**C**) calculated based on 95% BCI. The 20% threshold exceedance probability map (**D**) is also shown for the binomial model and also the uncertainty measured as the standard deviation of the predicted posterior prevalence for the binomial (**E**) and the Type I zero inflated binomial model (**F**). The data location is indicated by ‘+’ on the map showing uncertainty influenced by presence of the data. The magnitude of uncertainty is higher for the regular binomial model compared to the Type I zero inflated binomial model. The administrative borders are from the Global Administrative Areas (GADM) database (available at: https://gadm.org/maps.html).
