# Supplementary material for: Geospatial modeling of pre-intervention nodule prevalence of Onchocerca volvulus in Ethiopia as an aid to onchocerciasis elimination
Source: PLoS Negl Trop Dis. 2022 Jul 18;16(7):e0010620. doi: 10.1371/journal.pntd.0010620 (PMC9333447; doi:10.1371/journal.pntd.0010620)
Supplement: S6 Fig — The country boundary for the prediction region and the location of the observations (blue points) are shown on the triangulation mesh. The finer mesh is within the country boundary, while the coarser mesh is present outside the country boundary in the buffer region. The administrative borders are from the Global Administrative Areas (GADM) database (available at: https://gadm.org/maps.html). (DOCX) [file pntd.0010620.s010.docx]

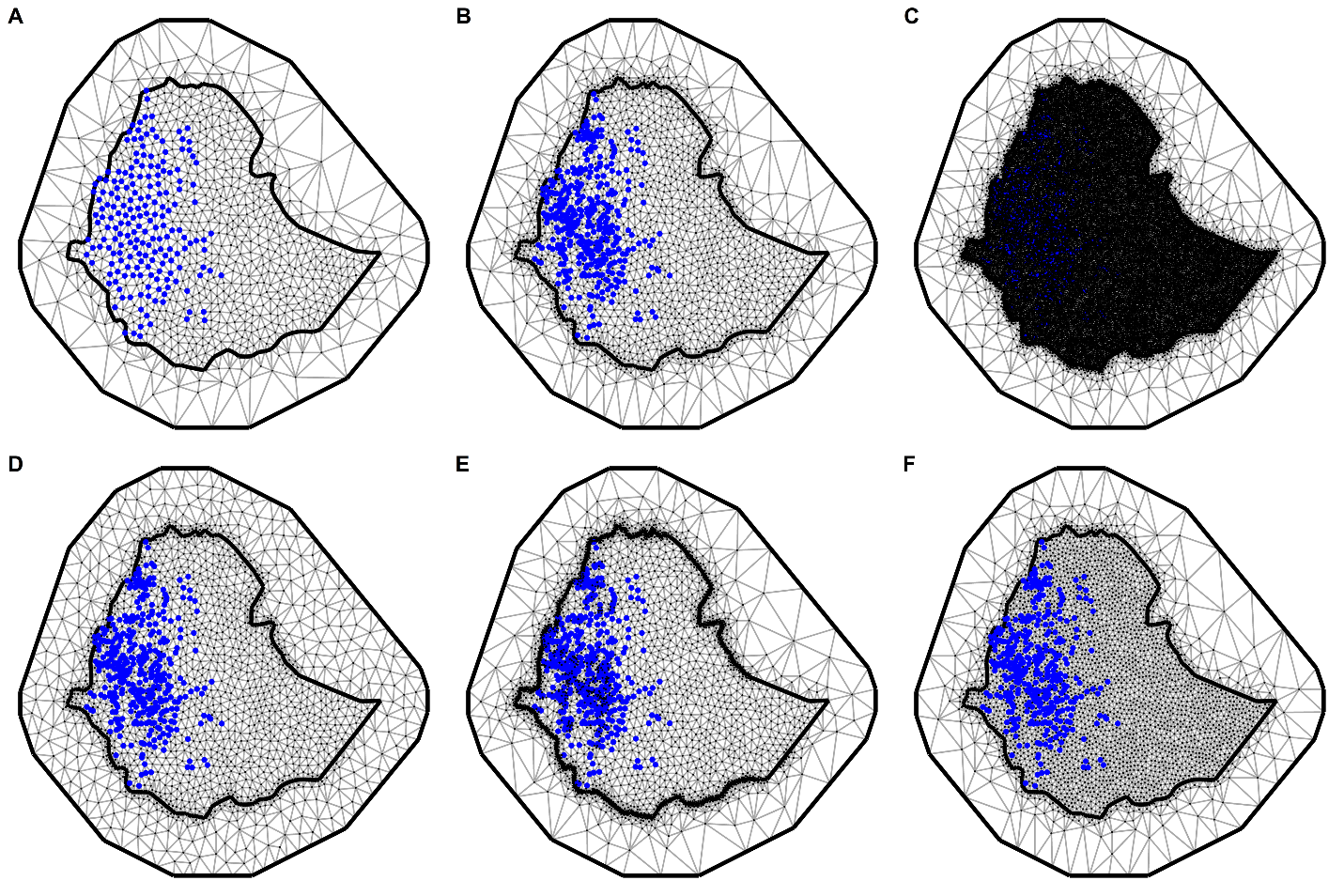


**S6 Fig. Different triangulated SPDE meshes considered for the analysis.** The country boundary for the prediction region and the location of the observations (blue points) are shown on the triangulation mesh. The finer mesh is within the country boundary, while the coarser mesh is present outside the country boundary in the buffer region. The administrative borders are from the Global Administrative Areas (GADM) database (available at: https://gadm.org/maps.html).
