# Supplementary material for: Geospatial modeling of pre-intervention nodule prevalence of Onchocerca volvulus in Ethiopia as an aid to onchocerciasis elimination
Source: PLoS Negl Trop Dis. 2022 Jul 18;16(7):e0010620. doi: 10.1371/journal.pntd.0010620 (PMC9333447; doi:10.1371/journal.pntd.0010620)
Supplement: S7 Fig — (A) Pearson correlation coefficient and (B) root mean square error (RMSE) were calculated between the predicted and the observed prevalence of each validation set during each cross-validation run. Model 0 is the model with only intercept and the spatial field, model 1 consists of six variables (slope, isothermality, precipitation seasonality, normalized difference vegetation index (NDVI), population density, and distance to the nearest river) with intercept and the spatial field, and model 2 consists of everything in model 1 with two additional variables (flow accumulation and soil moisture). (DOCX) [file pntd.0010620.s011.docx]

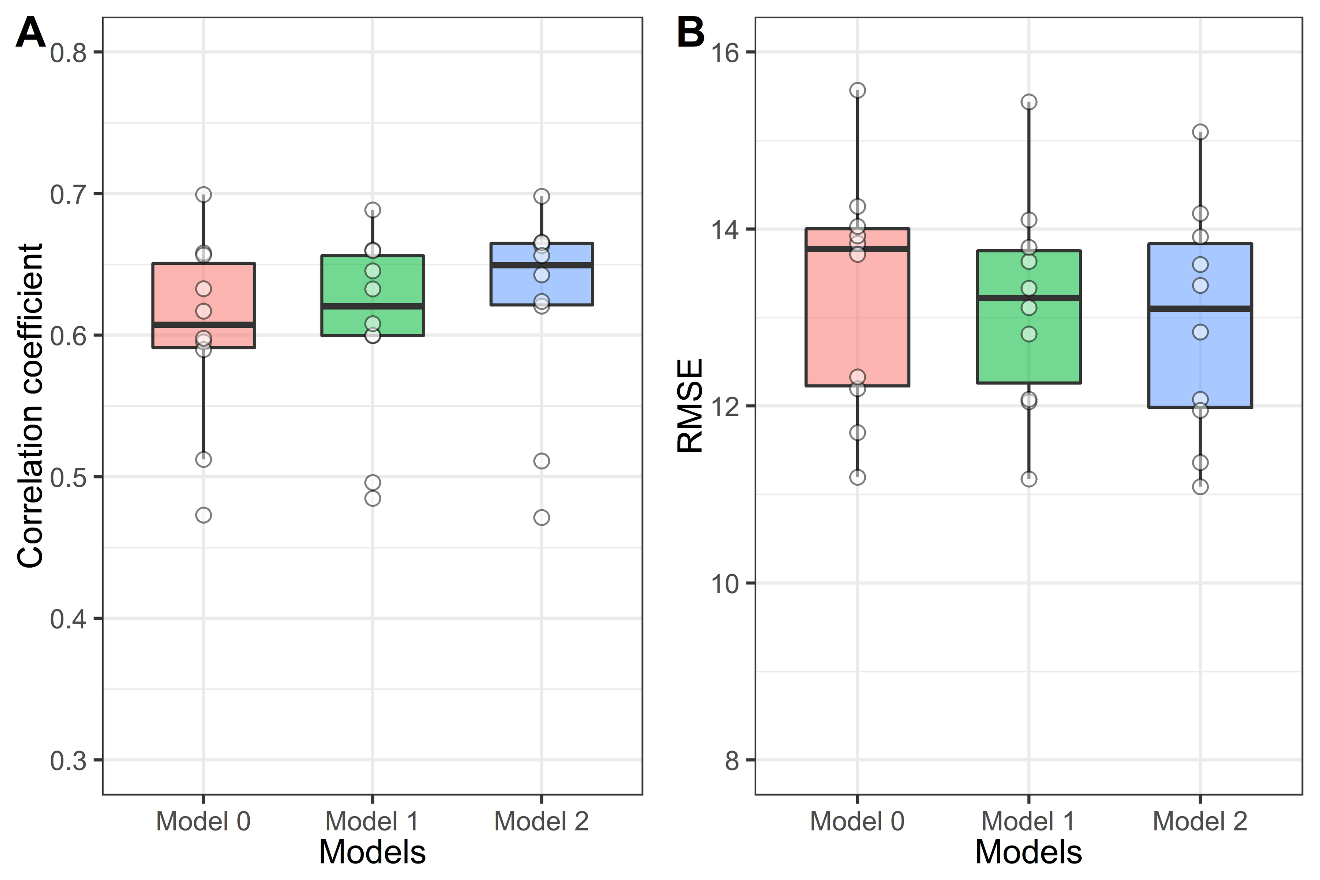


**S7 Fig. Boxplot showing cross-validation statistics obtained from 10-fold cross-validation from the three different geostatistical models.** (A) Pearson correlation coefficient and (B) root mean square error (RMSE) were calculated between the predicted and the observed prevalence of each validation set during each cross-validation run. Model 0 is the model with only intercept and the spatial field, model 1 consists of six variables (slope, isothermality, precipitation seasonality, normalized difference vegetation index (NDVI), population density, and distance to the nearest river) with intercept and the spatial field, and model 2 consists of everything in model 1 with two additional variables (flow accumulation and soil moisture).
