# Supplementary material for: Geospatial modeling of pre-intervention nodule prevalence of Onchocerca volvulus in Ethiopia as an aid to onchocerciasis elimination
Source: PLoS Negl Trop Dis. 2022 Jul 18;16(7):e0010620. doi: 10.1371/journal.pntd.0010620 (PMC9333447; doi:10.1371/journal.pntd.0010620)
Supplement: S8 Fig — The spatial field is higher in western Ethiopia. While the spatial field is lower in eastern Ethiopia, the standard deviation of the spatial field is higher. The administrative borders are from the Global Administrative Areas (GADM) database (available at: https://gadm.org/maps.html). (DOCX) [file pntd.0010620.s012.docx]

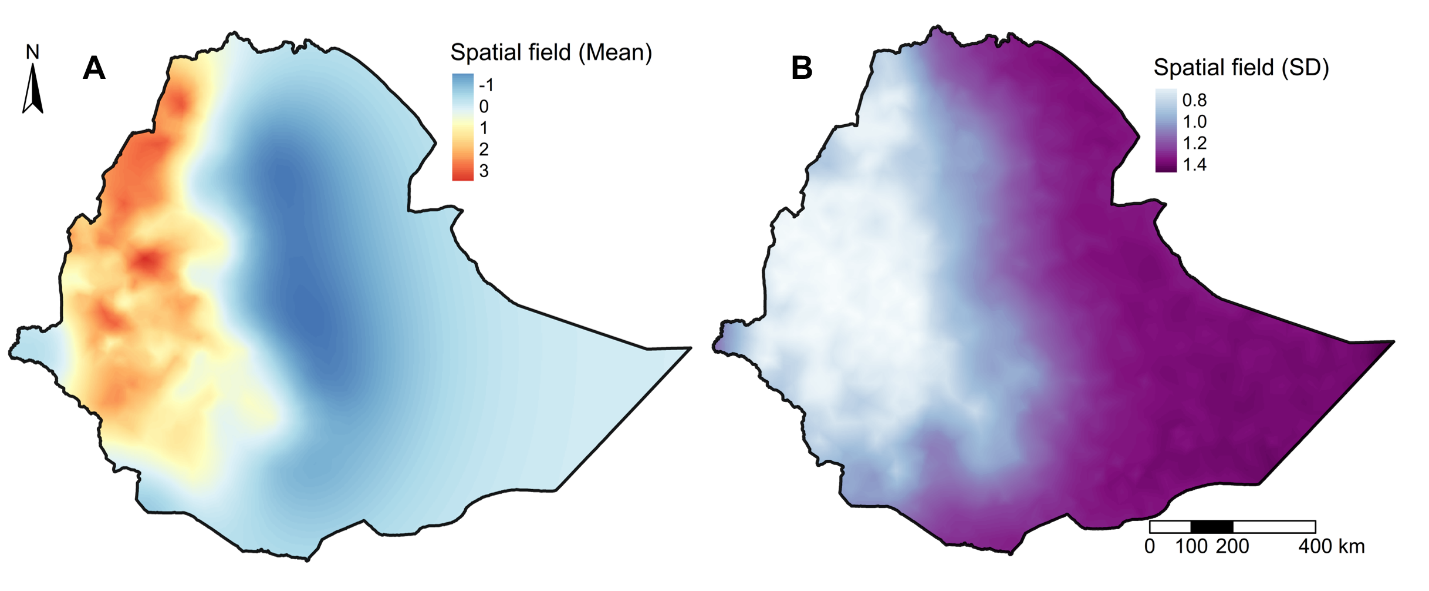


**S8 Fig. The spatial field's posterior mean (A) and standard deviation (B) from the stochastic partial differential equation (SPDE) mesh.** The spatial field is higher in western Ethiopia. While the spatial field is lower in eastern Ethiopia, the standard deviation of the spatial field is higher. The administrative borders are from the Global Administrative Areas (GADM) database (available at: https://gadm.org/maps.html).
