# Supplementary material for: Geospatial modeling of pre-intervention nodule prevalence of Onchocerca volvulus in Ethiopia as an aid to onchocerciasis elimination
Source: PLoS Negl Trop Dis. 2022 Jul 18;16(7):e0010620. doi: 10.1371/journal.pntd.0010620 (PMC9333447; doi:10.1371/journal.pntd.0010620)
Supplement: S9 Fig — The dashed line is the expectation for perfect correlation. The Pearson correlation coefficient and associated p-value are shown on the bottom left of the plot. Points are colored by the absolute difference between the observed and the predicted prevalence. (DOCX) [file pntd.0010620.s013.docx]

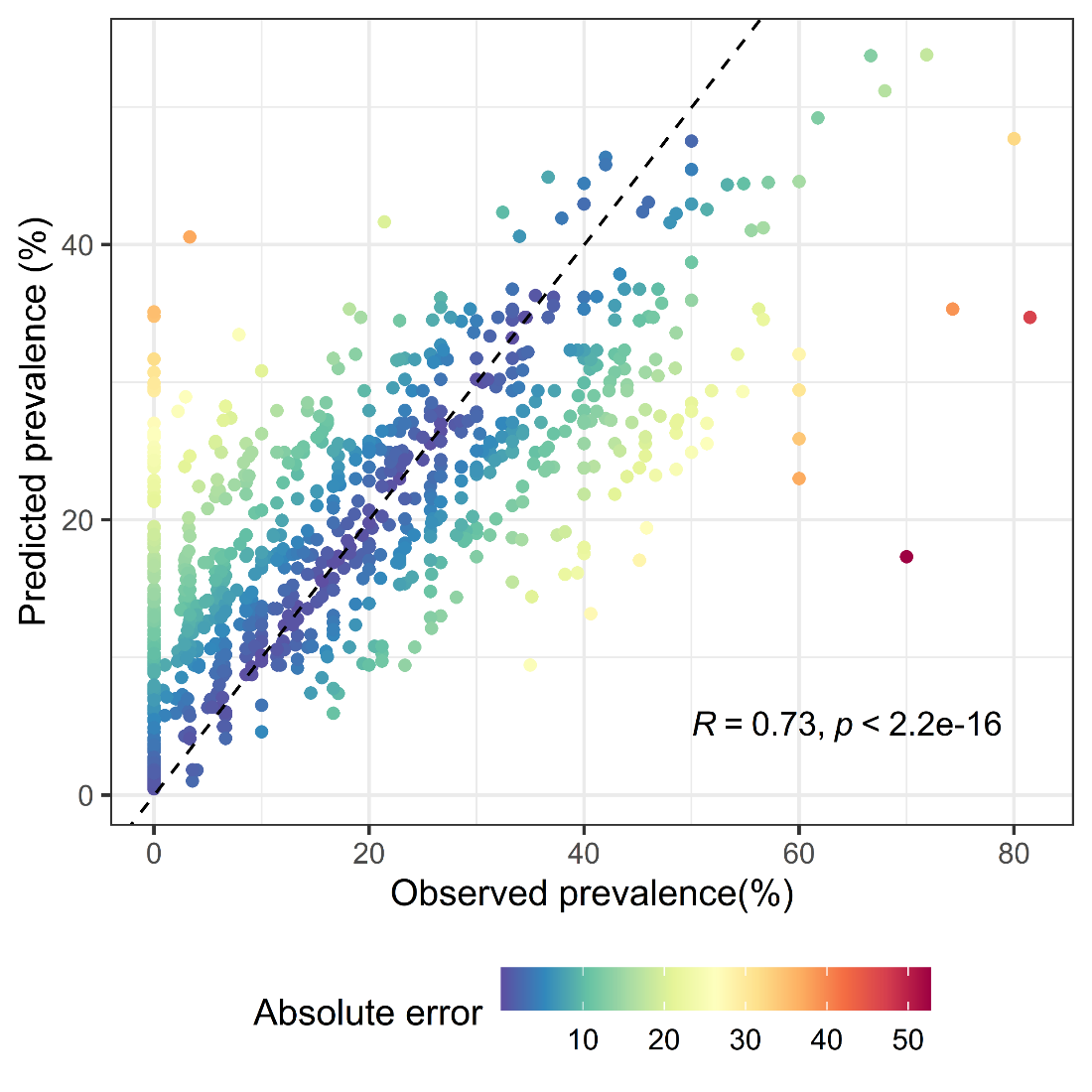


**S9 Fig. Correlation between the observed and predicted prevalence.** The dashed line is the expectation for perfect correlation. The Pearson correlation coefficient and associated p-value are shown on the bottom left of the plot. Points are colored by the absolute difference between the observed and the predicted prevalence.
