# Supplementary material for: Geospatial modeling of pre-intervention nodule prevalence of Onchocerca volvulus in Ethiopia as an aid to onchocerciasis elimination
Source: PLoS Negl Trop Dis. 2022 Jul 18;16(7):e0010620. doi: 10.1371/journal.pntd.0010620 (PMC9333447; doi:10.1371/journal.pntd.0010620)
Supplement: S10 Fig — (A) The mean of the estimated prevalence of all the pixels within the district level border and (B) the range of the estimated prevalence within the district, i.e. the difference between the highest prevalence pixel and the lowest prevalence pixel. The administrative borders are from the Global Administrative Areas (GADM) database (available at: https://gadm.org/maps.html). (DOCX) [file pntd.0010620.s014.docx]

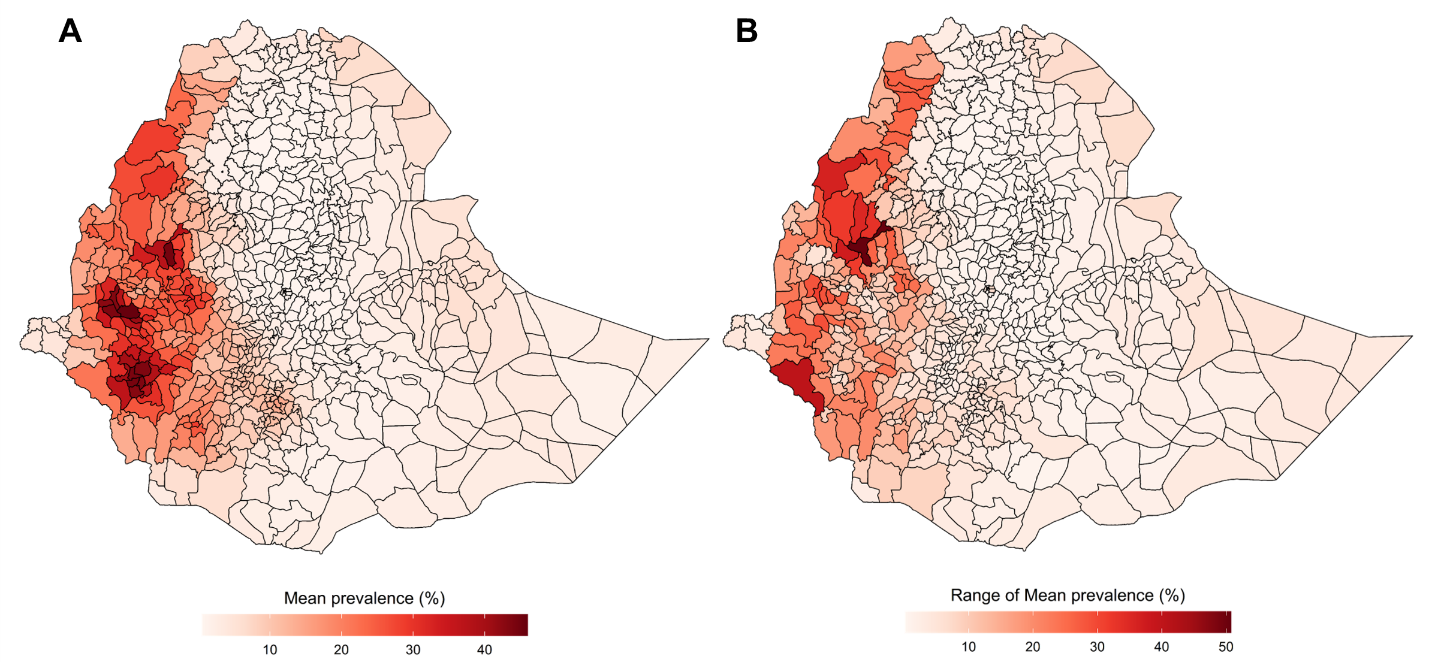


**S10 Fig. The aggregated mean prevalence and range of the estimated mean prevalence within Ethiopian districts.** (A) The mean of the estimated prevalence of all the pixels within the district level border and (B) the range of the estimated prevalence within the district, i.e. the difference between the highest prevalence pixel and the lowest prevalence pixel. The administrative borders are from the Global Administrative Areas (GADM) database (available at: https://gadm.org/maps.html).
