# Supplementary material for: Geospatial modeling of pre-intervention nodule prevalence of Onchocerca volvulus in Ethiopia as an aid to onchocerciasis elimination
Source: PLoS Negl Trop Dis. 2022 Jul 18;16(7):e0010620. doi: 10.1371/journal.pntd.0010620 (PMC9333447; doi:10.1371/journal.pntd.0010620)
Supplement: S11 Fig — The curve was fitted using the gam smoothing function available in the ggplot2 package for the purpose of visualization. The shaded region around the curve represents the 95% confidence interval. NDVI was rescaled from 0 to 100. NDVI: Normalized Difference Vegetation Index. (DOCX) [file pntd.0010620.s015.docx]

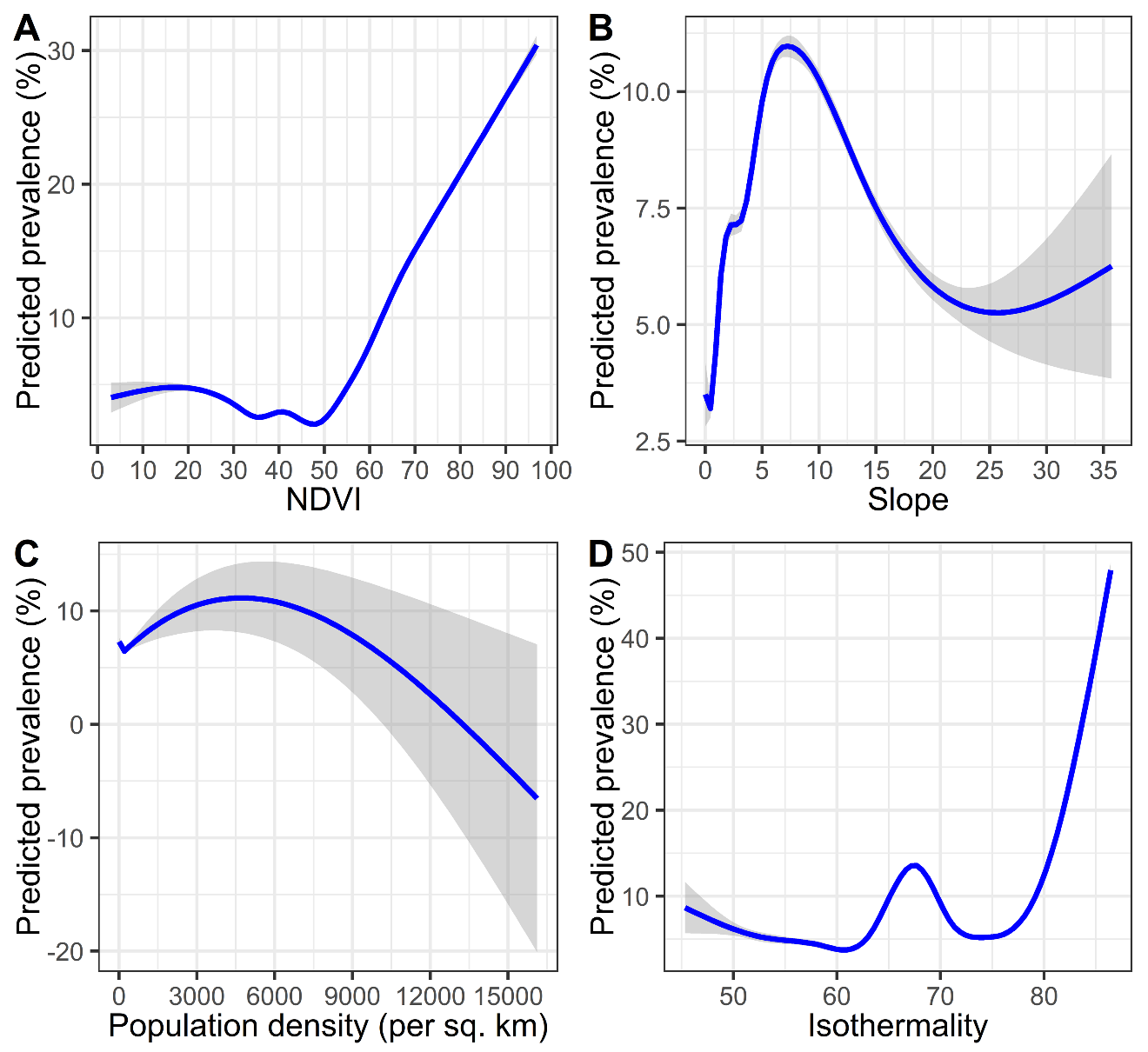


**S11 Fig. The relationship between the predicted posterior mean prevalence and the non-significant environmental and socio-demographic covariates in the regression model.** The curve was fitted using the *gam* smoothing function available in the *ggplot2* package for the purpose of visualization. The shaded region around the curve represents the 95% confidence interval. NDVI was rescaled from 0 to 100. NDVI: Normalized Difference Vegetation Index.
